# Biomimetic Mineralization of Keratin Scaffolds for Enamel Regeneration

**DOI:** 10.1002/adhm.202502465

**Published:** 2025-08-12

**Authors:** Sara Gamea, Elham Radvar, Dimitra Athanasiadou, Ryan Lee Chan, Giacomo De Sero, Ecaterina Ware, Sunie Kundi, Avir Patel, Shwan Horamee, Shuaib Hadadi, Mads Carlsen, Leanne Allison, Roland Fleck, Ka Lung Andrew Chan, Avijit Banerjee, Nicola Pugno, Marianne Liebi, Paul T Sharpe, Karina Carneiro, Sherif Elsharkawy

**Affiliations:** ^1^ Centre for Oral, Clinical, and Translational Sciences, Faculty of Dentistry Oral & Craniofacial Sciences King's College London London SE1 9RT UK; ^2^ Faculty of Dentistry Department of Restorative Dentistry Tanta University Tanta 31111 Egypt; ^3^ Faculty of Dentistry University of Toronto Toronto Ontario M5G 1G6 Canada; ^4^ Department of Physics Chalmers University of Technology Gothenburg 412 96 Sweden; ^5^ Institute of Biomedical Engineering University of Toronto Toronto Ontario M5S 3G9 Canada; ^6^ Laboratory for Bioinspired Bionic Nano Meta Materials & Mechanics Department of Civil Environmental and Mechanical Engineering University of Trento Trento 38123 Italy; ^7^ Department of Materials Royal School of Mines Imperial College London London SW7 2AZ UK; ^8^ Department of Chemistry King's College London London SE1 1DB UK; ^9^ Photon Science Division Paul Scherrer Institute Villigen 5232 Switzerland; ^10^ Centre for Ultrastructural Imaging New Hunts House Guys Campus King's College London London SE1 1UL UK; ^11^ Institute of Pharmaceutical Science King's College London London SE1 9NH UK; ^12^ School of Engineering and Materials Science Queen Mary University of London Mile End Road London E1 4NS UK; ^13^ Institute of Materials Ecole Polytechnique Fédérale de Lausanne (EPFL) Lausanne 1015 Switzerland; ^14^ Centre for Craniofacial and Regenerative Biology Faculty of Dentistry Oral & Craniofacial Sciences King's College London London SE1 9RT UK; ^15^ Prosthodontics Department Dental Directorate Guy's and St Thomas' NHS Trust London SE1 9RT UK

**Keywords:** biomineralization, enamel, regenerative dentistry, spherulites, white spot lesion

## Abstract

Biomimetic protein‐based platforms, with their hierarchical networks and optimal mechanical properties, show promising potential for hard tissue regeneration, including dental enamel. However, achieving aligned enamel‐like apatite nanocrystals from organic matrices remains challenging. A simple organic‐based approach to re‐create the hierarchical enamel structure using water‐based keratin films is reported. These films assemble via disulfide bridging into a fibrous organic network and birefringent spherulitic construction of predominant ordered β‐sheet conformation. The flexible structure of keratin templates facilitates rearrangement of the secondary structures into α‐helices upon mineralization, guiding the ordered growth of apatite nanocrystals. This system has shown potential in repairing early defective dental enamel lesions, restoring both optical appearance and mechanical properties. This study offers a promising, simple, and clinically‐friendly method for developing novel protein‐based matrices for hard tissue regeneration from naturally abundant sources.

## Graphical abstract

Keratin‐based films guide biomimetic enamel remineralization by promoting organized hydroxyapatite growth under physiological conditions. Advanced biophysical characterization confirms keratin's structural adaptability and mineral ions‐binding affinity, supporting mineral nucleation and hierarchical crystal assembly. This study establishes keratin as a promising, sustainable platform for functional enamel regeneration, offering a clinically translatable approach for repairing demineralized dental enamel lesions and restoring enamel architecture.

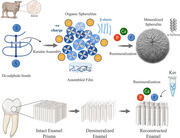

## 1 Introduction

Dental caries (tooth decay) is one of the most prevalent diseases globally, presenting the largest impact on advanced deterioration in oral health. According to the Global Burden of Disease 2019, untreated dental caries in permanent teeth is the most common health condition, with a prevalence of ≈2 billion cases.^[^ ^1^, ^2^ ^]^ Mature enamel is an acellular hard tissue that cannot regenerate if mineral loss occurs due to mechanical, chemical, or biological damage.^[^ ^3^ ^]^ If caries is left untreated, the disease progresses with adverse impacts on the patient, leading to eventual irreversible destruction of the dental tissues. This creates economic challenges due to the increased healthcare costs required to treat the consequences.^[^ ^4^ ^]^ Therefore, repairing the earliest signs of tissue damage caused by the caries process at the tooth enamel surface is key in developing biological functional systems to restore the native enamel structure and functionality.^[^ ^5^ ^]^

Biomimetic strategies for enamel repair have shown promise but still face significant limitations. Shao et al.,^[^ ^6^ ^]^ introduced a material composed of calcium phosphate ion clusters that demonstrated physical and mechanical recovery of damaged enamel. Nevertheless, only limited enamel thickness was regenerated, along with questionable biocompatibility in vivo. Furthermore, the Kotov group^[^ ^7^ ^]^ managed to create a multiscale mineralized architecture that resembled the enamel structure using a zinc oxide composite. However, the clinical applicability of this technique is challenging due to the complicated fabrication process. Oldak and co‐workers^[^ ^8^ ^]^ designed a peptide‐based hydrogel by incorporating amelogenin‐derived peptides with chitosan to treat incipient carious lesions. Even though this hydrogel enabled the growth of enamel‐like hydroxyapatite (HAp) crystals with increased biomechanical properties, the development of distinctive hierarchical apatite nanocrystals was not achieved. Another model of induced protein‐based mineralization is a study by Elsharkawy et al.,^[^ ^9^ ^]^ who reported a protein‐mediated mineralization process based on a tunable organic matrix using elastin‐like recombinamers. The process stimulated the hierarchical growth of aligned nanocrystals with enhanced mechanical properties. Nonetheless, the technique required non‐aqueous solvents and crosslinkers. Therefore, it has limited scalability. Additionally, Wang et al.^[^ ^10^ ^]^ developed an amyloid amelogenin analogue which promoted HAp crystal growth, forming a structured material with mechanical properties, yet precise control over HAp crystallization proved challenging.

Keratin is a protein that has garnered attention for its potential biomedical applications, particularly in regenerative medicine.^[^ ^11^, ^12^, ^13^ ^]^ Keratin, particularly α‐keratin found in wool, exhibits a complex hierarchical structure that enables its self‐assembly and contributes to its mechanical strength and stability. The organic structure of keratin is characterized by its complex hierarchy and dense network of covalent and noncovalent interactions, including its ionic, hydrogen, and disulfide bonds. Covalent disulfide bonds, formed through cystine cross‐links between cysteine residues, play a crucial role in stabilizing the polypeptide chains, thereby enhancing the fiber's mechanical integrity. Noncovalent interactions further facilitate keratin self‐assembly into highly ordered, mechanically robust structures essential for its biological function and resistance to chemical and enzymaticdegradation.^[^ ^14^, ^15^ ^]^ Although some studies have highlighted the structural and functional significance of keratin in tooth enamel,^[^ ^16^, ^17^ ^]^ research investigating its biomedical applications in enamel repair and regeneration remains limited.

In this study, water‐based, self‐crosslinking keratin films able to facilitate dynamic conformational changes and provide templates for hierarchical mineralization were developed. These keratin films are reported to optimize their conformation upon mineralization and control the nucleation and growth of mineralized structures, therefore infiltrating early enamel carious lesions to restore their intrinsic crystallographic structure, cosmetic appearance, and mechanical capabilities. This negates the need for invasive operative intervention and paves the way for a paradigm shift in the clinical management of early carious lesions.

## 2 Results and Discussion

### 2.1 Keratin Extraction and Characterization

In an attempt to rearrange the reduced bonds and design supramolecular organic films for biomedical and dental applications, natural keratins were extracted from wool fibers under reducing conditions (Illustrated in Figure S1A,B, Supporting Information), facilitating the cleavage of disulfide bonds.^[^ ^15^ ^]^ The extract was then purified and freeze‐dried, and the protein concentration was confirmed via Bicinchoninic acid (BCA) assay (Figure S1C, Supporting Information). The protein composition and molecular weight were verified by sodium dodecyl sulphate‐polyacrylamide gel electrophoresis analysis (SDS‐PAGE) (Figure S1D, Supporting Information), revealing low‐sulfur (LS) type I (45–50 kDa) and type II (55–60 kDa) keratins, high‐sulfur proteins (HSPs) (12–28 kDa), and high‐glycine‐tyrosine proteins (HGTs) (7–12 kDa). Our pre‐designed keratin films utilized these different types of keratins to optimize their functionality. For example, LS keratins, specialized for the formation of intermediate filaments, provide mechanical strength to the matrix. HSPs, rich in cysteines, create disulfide crosslinks that enhance insolubility, structural stability, and resistance to enzymatic degradation.^[^ ^18^ ^]^ HGTs found in keratin‐associated proteins of hair and wool likely contribute to matrix cohesion and durability.^[^ ^19^ ^]^

Extraction was further confirmed by Attenuated Total Reflection‐Fourier Transform Infrared Spectroscopy (ATR‐FTIR) (Figure S1E, Supporting Information). Characteristic peaks of protein secondary structures were observed: amide A at 3248 cm_-__1_ (N─H stretching), amide I at 1623 cm_-__1_ (C═O stretching), amide II at 1457 cm_-__1_ (N─H bending and C─N stretching), and amide III at 1250 cm_-__1_ (C─N, C─O stretching, and N─H bending).^[^ ^20^ ^]^ Peaks at 1063 cm_-__1_ indicated oxidized cysteine residues (SO_2_, SO, S═O), a by‐product of disulfide bond cleavage during extraction.

Finally, Liquid Chromatography with tandem Mass Spectrometry (LC‐MS/MS) analysis of SDS‐PAGE bands confirmed keratin subtypes. Band A (55–60 kDa) predominantly contains keratin type II microfibrillar proteins (Figure S1F, Supporting Information), including Component 5 (55 kDa; P25691), Component 7C (54 kDa; P15241), and a Fragment (13 kDa; P02539). Similarly, Band B (35–50 kDa) predominantly contained keratin, type I microfibrillar proteins (Figure S1G, Supporting Information), including Type I microfibrillar (47.6 kDa; P25690) and Type I microfibrillar, component 8C‐1 (Molecular Weight 48 kDa; P02534).

### 2.2 Biophysical Characterization of Keratin in Solution

Circular dichroism (CD) and dynamic light scattering (DLS) were used to assess keratin's secondary structure and supramolecular behavior across pH levels. CD spectra (Figure S2A, Supporting Information) showed characteristic α‐helix signals (negative peaks at 208 and 220 nm; π→π* transition at ≈190 nm) at all pH levels. With increasing pH, shifts toward β‐sheet features were observed (negative band at −216 nm; positive band at 195–200 nm).^[^ ^21^ ^]^ Deconvolution (Figure S2B, Supporting Information) confirmed a mixture of α‐helix, β‐sheet, random coil, and turn structures. Increasing pH from 7 to 11 reduced α‐helix content (from 10.3 ± 1.25% to 6.9 ± 0.7%) and increased random coil (from 25.8 ± 1.65% to 33.03 ± 0.2), while β‐sheet and turns remained relatively constant. These transitions support keratin's intrinsic disorder‐like behavior, similar to intrinsically disordered proteins (IDPs), which adopt more folded structures under favorable conditions (e.g., pH), likely due to hydrophobic collapse and molecular rearrangement.^[^ ^22^, ^23^ ^]^ DLS analysis showed that keratin solutions were negatively charged at all pH values (Figure S2C, Supporting Information), with increasing negativity at lower pH, likely due to glutamic and aspartic acid residues, which are thought to play a critical role in promoting the formation and organization of HAp crystals and enhancing strong calcium‐binding affinity.^[^ ^24^, ^25^ ^]^ Hydrodynamic radius increased from 53.8 nm at pH 7 to ≈87.5 nm at pH 11 (Figure S2D, Supporting Information), indicating pH‐induced aggregation. Upon the addition of calcium ions, CD deconvolution (Figure S2E,F, Supporting Information) revealed increased α‐helix and decreased β‐sheet content across all pH, alongside reduced zeta potential and increased hydrodynamic size and polydispersity (Figure S2G,H, Supporting Information), which suggests keratin‐calcium binding. These results coincide with a study by Lu et al.^[^ ^26^ ^]^ on amelogenin, which suggested that the protein prevails in two forms: either in a disordered form and mobile or organized in a β‐sheet conformation with less mobility when they are present in the form of nanospheres. Implying that these structural organizations complement each other and that the β‐sheet structure is poised to interact with HAp.^[^ ^27^ ^]^

Isothermal titration calorimetry (ITC) was also employed to characterize the thermodynamics of calcium ion binding to keratin. Titration of CaCl_2_ into keratin produced exothermic heat signals with progressively decreasing amplitude, consistent with progressive saturation of available binding sites (Figure S2I, Supporting Information). The integrated binding isotherm revealed a dissociation constant (Kd) of 2.85 mM, indicating moderate affinity between keratin and calcium ions (Figure S2J, Supporting Information). The stoichiometry was determined to be 1.00, demonstrating that one mol of calcium ion binds per mol of keratin monomer, indicative of complete occupancy of available binding sites. The binding interaction was exothermic, with an enthalpy change (ΔH) of −25.0 ± 2.34 kcal mol^−1^ and a Gibbs free energy change (ΔG) of −3.44 kcal mol^−1^, confirming that the interaction is spontaneous and favors the bound state under the tested conditions. The entropy component (−TΔS) was calculated to be 21.6 kcal mol^−1^, indicating a negative entropy change associated with the binding process. These results suggest that the binding of calcium ions to keratin is predominantly enthalpy‐driven, likely due to the formation of specific interactions such as ionic coordination with acidic residues, which compensate for the entropic penalty associated with increased system order upon binding.^[^ ^28^, ^29^, ^30^ ^]^ The observed thermodynamic signature aligns with the proposed role of keratin in biomineralization, where its capacity to coordinate calcium ions can facilitate nucleation events under physiological conditions. The moderate binding affinity and enthalpy‐driven nature of the interaction indicate that keratin can effectively localize calcium ions while allowing for reversible interactions essential for dynamic mineralization processes, supporting its potential as a biomimetic scaffold in hard tissue repair applications.

### 2.3 Keratin Films Fabrication, Optimization, and Reconstitution of the Di‐Sulfide Bonds

Keratin film formation involves self‐assembly where polar side chains interact with water while nonpolar residues form a hydrophobic core.^[^ ^31^ ^]^ Hydrogen bonding between water molecules and keratin's amino and carboxyl groups further stabilizes the structure.^[^ ^32^, ^33^ ^]^ To enhance film properties, triethylene glycol dimethacrylate (TEGDMA) was incorporated, as methacrylate groups can react with keratin's cysteine thiol groups via thiol‐Michael addition, forming covalent crosslinks that strengthen the network.^[^ ^34^, ^35^ ^]^ This modification can improve mechanical stability, flexibility, and reduce brittleness while enhancing resistance to enzymatic degradation.^[^ ^36^ ^]^ Additionally, TEGDMA allows tuning of film porosity and stiffness, increasing suitability for long‐term biomedical applications.^[^ ^37^ ^]^

**Figure 1:**
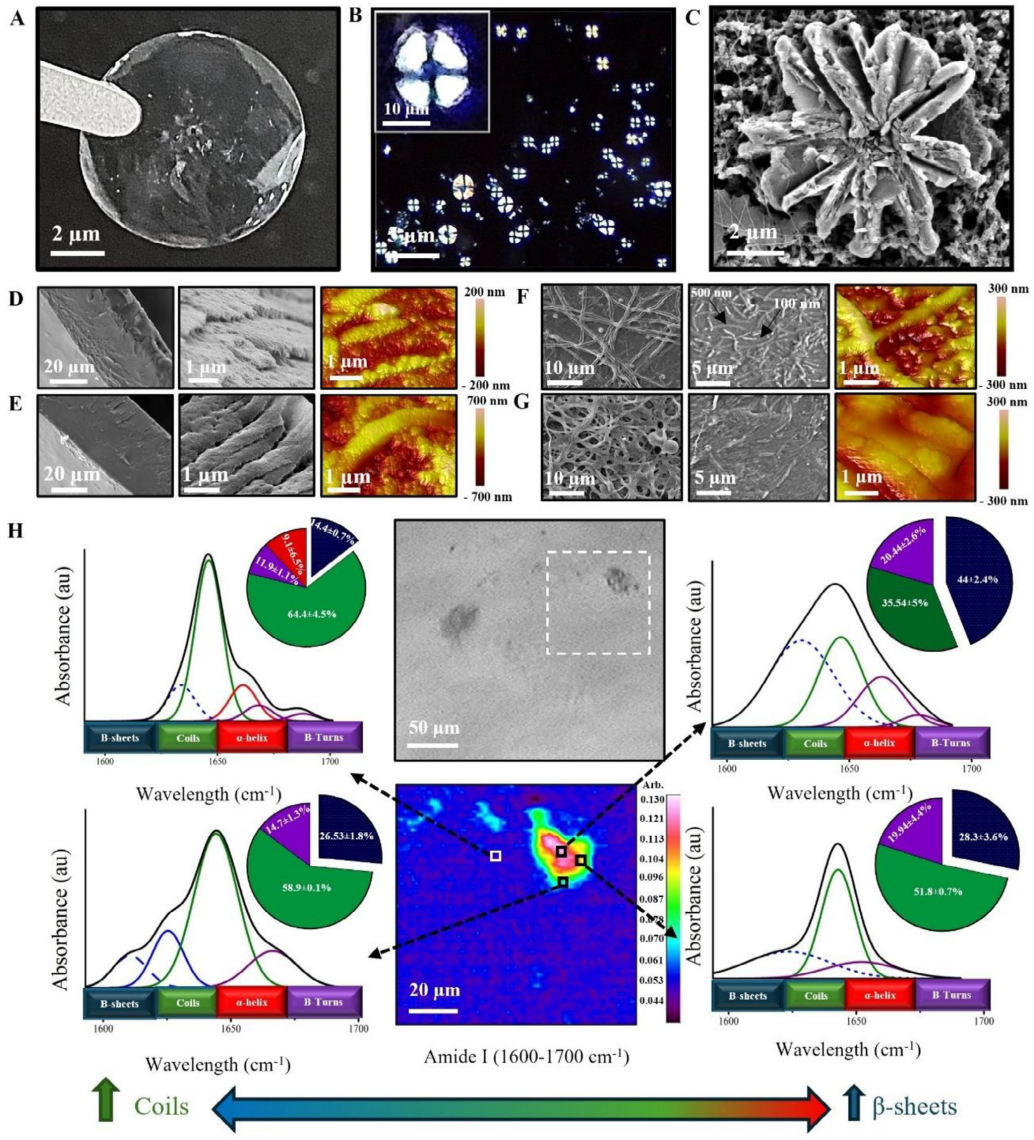
Keratin organic matrix assembly and secondary structure characterization. A) Image showing a keratin film before mineralization, after rinsing in water for 3 days and dehydrating. B) PLM image of an organic keratin film depicting organic spherulites on its surface, ^*^inset showing high magnification of a keratin spherulite with the characteristic birefringent Maltese‐cross appearance. C) SEM showing a fan‐like structure within the keratin film before mineralization. Self‐assembly of the keratin films; D) Ker_5_, and E) Ker_10_, F) Ker_5_TE_1_, G) Ker_10_TE_1_. H) FTIR imaging of an organic film demonstrating an area where a spherulite was imaged with a light microscope. FTIR amide I (1600–1700 cm^−1^) corresponding to four different areas of the film were outlined on the chemical map and captured, and their corresponding secondary structure deconvolution percentages are presented in the relative pie charts.

Keratin films were fabricated by solubilizing the lyophilized keratin in ultra‐pure water (UPW) under varying concentrations, pH, and crosslinking conditions (illustrated in Figure S3A, Supporting Information), followed by air drying to promote self‐crosslinking via disulfide bond reformation, rendering the films insoluble (**Figure** **1**A). A structured pilot optimization assessed film ease of handling, collapse potential, brittleness, and transparency using a standardized scoring system (Tables S1 and S2, Supporting Information). Self‐crosslinked films at pH 7 and 11 demonstrated high transparency, with increasing keratin concentrations (3–10% w/v) improving handling and reducing brittleness, particularly at pH 7. TEGDMA‐crosslinked films further highlighted the influence of pH and crosslinking density. At pH 7, increasing keratin concentration and TEGDMA content (0.04–0.8% w/v) enhanced flexibility and reduced collapse potential while increasing opacity. Films at pH 11 exhibited higher brittleness and collapse potential, indicating less favorable mechanical properties under these conditions. Overall, optimal formulations balancing transparency, mechanical stability, and structural integrity were identified and selected for subsequent mineralization and enamel repair studies.

To assess the progression of disulfide bond formation during film formation, Ellman's assay was performed at three time points on samples from different stages of keratin film preparation (Figure S3B, Supporting Information). The concentration of free cysteine, indicative of reduced disulfide bonds, was monitored. Samples solubilized in water initially exhibited higher free cysteine concentrations than crosslinked keratin samples, suggesting ongoing disulfide bond formation and reduced free thiols over time. The crosslinked keratin samples had small differences in the cysteine concentrations, demonstrating slower drying rates.

### 2.4 Keratin Films Self‐Assembly, Surface Morphology, And Conformation

After drying, keratin‐based films exhibited birefringent spherulites (1–10 µm) with a characteristic Maltese cross under a polarized light microscope (PLM) (Figure 1B). The abundance and organization of these crystalline spherulites increased with higher keratin concentrations (10% w/v), forming large clusters and aligned strands (Figure S4A,B, Supporting Information). These structures, densely packed within protein chains, contribute to enhanced mechanical strength and rigidity.^[^ ^38^ ^]^ Congo red staining of the keratin films demonstrated dendritic structures within the size range of the spherulites (Figure S4C, Supporting Information). Scanning electron microscopy (SEM) imaging revealed these spherulites as fan‐like structures (Figure 1C) alongside networks of nanospheres and microfibrils on the film surfaces (Figure 1D–G).

Microfibrillar networks were observed in both self‐crosslinked and TEGDMA‐crosslinked films, with fibril diameter increasing with keratin concentration (Figure 1D–G; Figure S4D–F, Supporting Information). In Ker_5_TE_1_ (5% w/v keratin with TEGDMA), decussating fibrils of ≈100 and ≈500 nm were observed (Figure 1F), while Ker_10_TE_1_ (10% w/v) displayed well‐aligned parallel fibrils (Figure 1G). A decrease in density of keratin films following crosslinking with TEGDMA was observed, which is likely attributed to its flexible, low‐molecular‐weight structure introducing spatial gaps, increasing porosity, and reducing packing density. Steric hindrance from TEGDMA's bulky groups and phase separation during crosslinking further contributed to this open microstructure.^[^ ^39^ ^]^ Atomic force microscopy (AFM) analysis of keratin solution before drying revealed keratin nanospheres in solution averaging 24.2 ± 5.3 nm at 5% w/v, increasing to 33.0 ± 6.9 nm at 10% w/v (Figure S5A,B, Supporting Information), with similar 20–40 nm structures persisting after drying (Figure 1D–G) These nanospheres are of interest due to their potential in enhancing mechanical stability and biomineralization, analogous to the role of amelogenin nanospheres in enamel formation that facilitate HAp nucleation and alignment.^[^ ^23^, ^40^, ^41^ ^]^

In order to understand the contribution of protein secondary structures in the formation of the organic keratin spherulites and their influence on the mineralization motifs, the Fourier Transform Infrared Spectroscopy (FTIR) imaging technique was employed. Amide I band deconvolution data were analyzed from the organic spherulite and its surroundings (Figure 1H). FTIR analyses revealed that the keratin film adopted mainly β‐sheets, as well as random coil structures. Moving away from the spherulite, the proportion of β‐sheets decreased (44 ± 2.4%, 28.3± 3.6%, 26.5± 1.8%, and 14.4 ± 0.7%), while the random coils increased (35.5 ± 5%, 51.8 ± 0.7%, 58.9 ± 0.1%, and 64.4 ± 4.5%), with α‐helix structures observed only in the surrounding area (9.11± 6.5%).

### 2.5 Mineral Nucleation and Crystallographic Characterization

The mineralization potential of the keratin films was assessed by incubating them in a supersaturated fluoride‐rich HAp solution at 37 °C. Similar to amelogenin, keratin shows a propensity to self‐assemble into nanoscale structures under specific conditions, indicating a comparable mechanism in keratin‐mediated mineralization. During a 7‐day mineralization period, keratin nanospheres initially aligned into ordered rows (Figure S5C,D, Supporting Information) before disassembling into smaller spheres that fused into mineralized apatite structures (Figure S5E,F, Supporting Information). These keratin nanospheres function as templates for HAp nucleation and growth, effectively mimicking the natural biomineralization process observed during enamel formation. These observations indicate that keratin nanospheres act as templates for HAp nucleation and growth, mimicking natural enamel biomineralization. The role of these nanospheres in mineralization complements keratin's other unique properties, including its negatively charged carboxylic acid groups and the β‐sheet ordered conformation within the organic matrix, which collectively regulate the growth and organization of HAp crystals.

**Figure 2:**
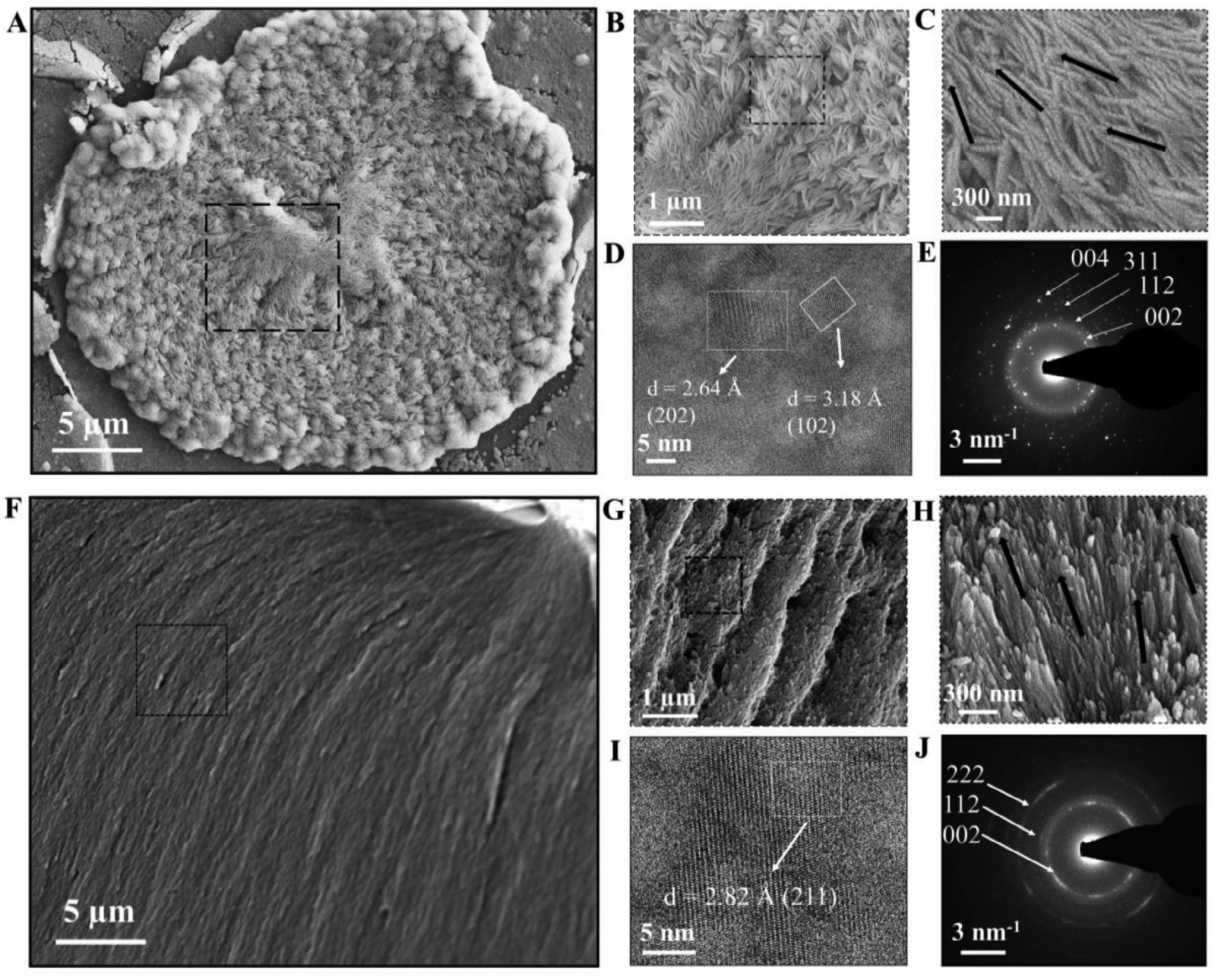
Microstructural characterization of mineralized spherulitic structures on keratin films at day 3. A) SEM image showing mineralized spherulitic structures exhibiting a spirally oriented morphology of assembled needle‐like mineral nanocrystals. B) Higher magnification SEM image displaying aligned, prism‐like formations within the spherulite, measuring 1.36 ± 0.33 µm in thickness and extending over tens of micrometers in length along the radial axis. C) Prisms composed of nanocrystals with average diameters of 83.0 ± 23.0 nm, radiating outward from the spherulite center toward its periphery. D) HR‐TEM image from a FIB milling lift‐out of a mineralized structure, illustrating apatite crystals from the film surface. E) SAED of the surface apatite crystals displaying polycrystallinity. F–H) SEM image of a mineralized keratin film cross‐section. (G,H) Higher magnification images showing elongated apatite nanocrystals with average diameters of 54.3 ± 27.2 nm, exhibiting an organized, parallel arrangement within the bulk. I) HR‐TEM image of bulk apatite crystals demonstrating growth orientation and crystal lattice d‐spacing J) SAED pattern of bulk apatite crystals within the mineralized layer, forming concentric diffraction patterns indicative of high structural ordering within the mineralized matrix.

SEM analysis of mineralized keratin films revealed spherulite‐like mineralized structures (Figure S6A, Supporting Information). On day one, smaller spherulites with few defined crystals were observed (Figure S6B, Supporting Information). PLM also indicated a limited number of spherulites with some platelet‐like crystals (Figure S6C, Supporting Information). By day two, apatite crystals began to develop (Figure S6D, Supporting Information), accompanied by a reduction in platelet‐like crystals and increased spherulite number as observed under PLM (Figure S6E, Supporting Information). On day three, mineralized spherulitic structures exhibited a spirally oriented morphology of assembled needle‐like mineral nanocrystals (**Figure** **2**A). These structures featured aligned, prism‐like formations measuring 1.36 ± 0.33 µm in thickness and extending over tens of micrometers in length (Figure 2B). These prisms comprised polycrystalline nanocrystals with average diameters of 83.0 ± 23.0 nm, radiating from the center of the spherulite toward its periphery (Figure 2C‐E). Within the bulk of the mineral, the prisms comprised elongated apatite nanocrystals with average diameters of 54.3 ± 27.2 nm, exhibiting a more organized, parallel arrangement and demonstrating concentric diffraction patterns (Figure 2F–J). By day 7, mineralized structures exhibited increased mineral deposition spreading over the film (Figure S7A, Supporting Information), which became denser and more needle‐like across the surface by day 10 (Figure S7B, Supporting Information). On day 14, larger merged spherulites with fine nanocrystalline matrices were evident (Figure S7C, Supporting Information). By day 30, extensive networks of radially oriented needle‐like crystals had formed, demonstrating progressive growth in size and coverage across the substrate (Figure S7D, Supporting Information), indicative of advanced mineralization over time.

**Figure 3:**
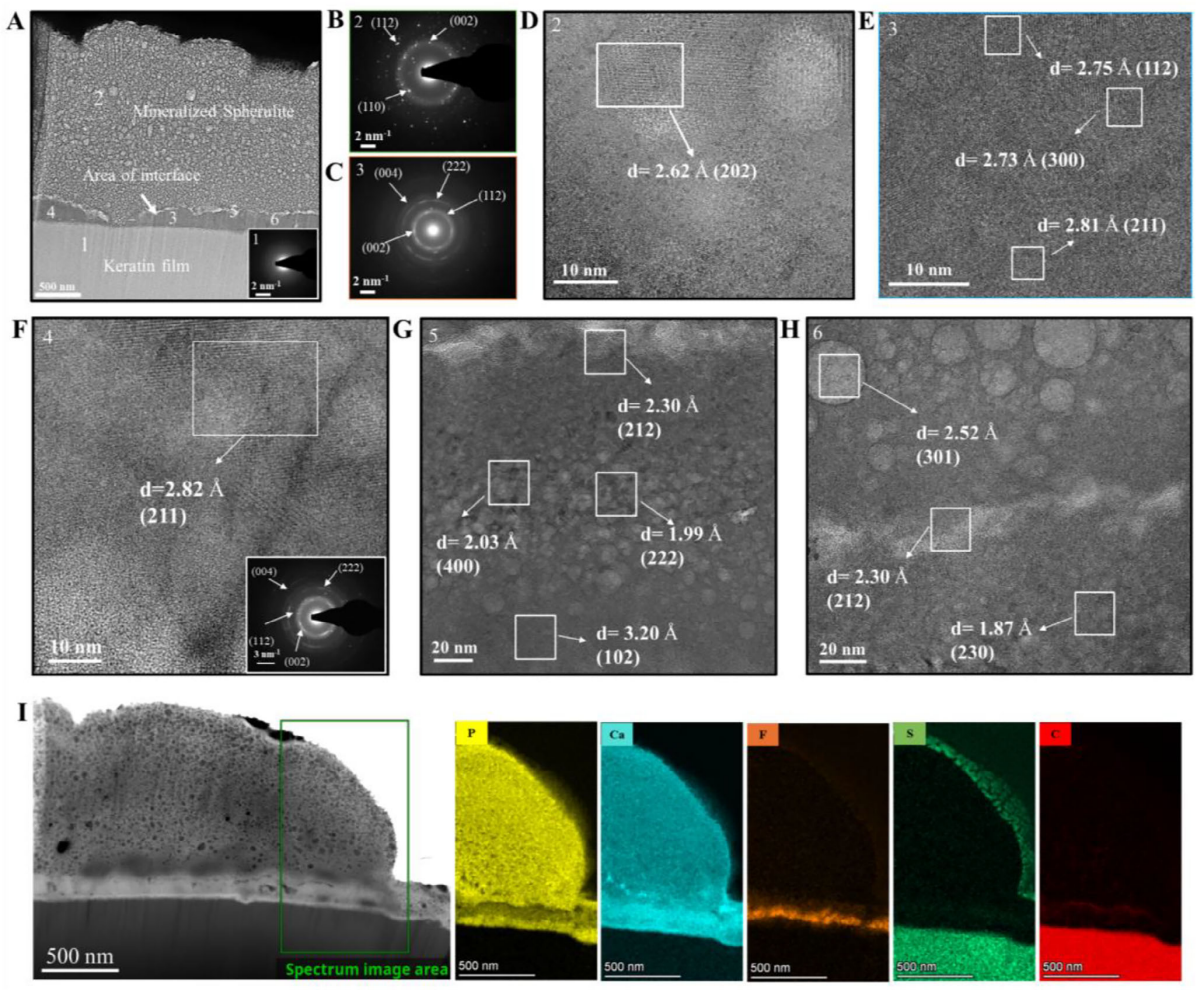
Crystal characterization of keratin induced mineralized structure. A) HR‐TEM images from a FIB milling lift‐out of a mineralized structure illustrating three different zones: Top mineralized zone, middle interface zone, and base organic zone, highlighting 6 different areas on the FIB lamella numbered from 1 to 6, ^*^inset showing SAED of the organic layer (1) revealing no crystallization pattern. B) SAED of the top mineralized spherulite zone demonstrating a poly‐crystalline pattern. SAED of the top and middle zones, respectively. C) SAED of middle zone (interface) demonstrating arching in the crystalline pattern, which indicates a higher degree of organization and could be an indication of preferential orientation of the crystals by the keratin. D–H) HR‐TEM of different areas on the FIB lamella numbered with respect to the numbers shown in A and demonstrating the crystal d‐spacing that confirms the apatite phase. I) Dark field TEM image of the FIB lamella with the corresponding EDX analysis displaying the chemical compound distribution in the three zones, where the top zone is made up of large amounts of calcium and phosphorus, while the interface constitutes mainly fluoride. The base is made up entirely of carbon, sulphur, and nitrogen.

Focused ion beam (FIB) milling (Figure S8, Supporting Information) followed by high‐resolution transmission electron microscopy (HR‐TEM) and selected area electron diffraction (SAED) revealed three distinct structural zones within the mineralized spherulite (**Figure** **3**A): top, interface, and base zones. The top zone exhibited polycrystalline circular structures with an average diameter of 22.6 ± 4.4 nm (Figure 3B). Within the interface layer, crystalline diffraction patterns exhibiting arching at 002, 112, and 222 planes were reported (Figure 3C), corresponding to the crystallographic planes of apatite.^[^ ^42^ ^]^ The density and size of these circular structures decreased progressively from the top toward the interface, where they became sparse (Figure 3D–H). Crystallite d‐spacing analysis validated the presence of apatite crystals on the spherulite surface. The arching observed within the interface layer likely reflects the texture and preferential orientation of the crystals within the bulk of the keratin, indicating a higher degree of organization. In contrast, the base zone consisted entirely of the keratin organic matrix without detectable crystalline structures (Figure 3A). Energy dispersive x‐ray analysis (EDX) (Figure 3I) showed that the top zone contained high levels of calcium and phosphorus, along with smaller amounts of sulfur, carbon, and fluoride. The interface zone was predominantly enriched in fluoride, while the base zone was composed mainly of carbon, sulfur, and nitrogen.

These findings suggest mineralization initiates at the interface, where proximity to keratin facilitates the formation of organized, fluoride‐rich apatite, while the top layer may contain trapped organic nanospheres acting as additional nucleation sites or partially crystallized amorphous calcium phosphate (ACP), consistent with prior observations^[^ ^43^ ^]^ who observed amelogenin organic signatures across the mineralized particulate, suggesting that proteins can become entrapped during HAp crystal aggregation and fusion.^[^ ^43^ ^]^ The presence of organic nanospheres may result from matrix entrapment during apatite growth or due to limited transformation of ACP to apatite in highly mineralized conditions, warranting further investigation under physiological conditions. The partial transformation of ACP in the outer mineralized layer underscores the essential role of the organic matrix in mediating apatite formation, likely due to the limited availability of nucleation sites. Overall, the keratin matrix directs mineral nucleation and crystal alignment, potentially by protein adsorption controlling growth along the c‐axis and modulating supersaturation, thereby mimicking natural biomineralization pathways in enamel formation. Future work should focus on functionalizing keratin with additional acidic residues to enhance nucleation site density and mineralization kinetics

### 2.6 Mineralization Kinetics of Keratin

The crystalline phase of the mineralized films over 30 days was then verified using Magic Angle Spin‐Nuclear Magnetic Resonance (MAS‐NMR). Fluorine‐19 (^19^F) MAS‐NMR spectra showed a sharp peak at −103 ppm on day 1, indicating the presence of fluorapatite (FAp). By day 3, an additional peak appeared at −108 ppm, corresponding to fluorite (CaF_2_), alongside the original peak at −103 ppm. This peak becomes more prominent in the keratin films by day 7, suggesting continued mineral maturation and coexistence of both phases within the films. (Figure S9A, Supporting Information). By Day 10, 14, and 30, the CaF_2_ peak became dominant, with a sharper and more defined signal, while the FAp peak at −103 ppm appeared as a shoulder, suggesting substantial CaF_2_ formation alongside residual FAp. Complementary ATR‐FTIR spectra of the mineralized keratin films over 30 days demonstrated progressive changes indicative of apatite formation and maturation on the keratin matrix (Figure S9B, Supporting Information). Distinct phosphate (PO_4_^3^_-_) vibrational bands were evident, including the ν3‴ antisymmetric stretching modes within the 1000–1100 cm^−1^ region and the ν1' symmetric stretching mode ≈960–965 cm^−1^. Over time, the intensities of these PO_4_^3−^ bands increased, particularly evident from Day 1 to 30, while the intensity of the protein amide bands decreased with time. This reflects a relative reduction in the organic content on the film surface as mineral deposition progressed. Additionally, the spectra displayed bands within the 1500–1400 cm^−1^ region, corresponding to the antisymmetric ν3 C─O stretching, and a band near 875 cm^−1^, corresponding to the ν2 vibration of carbonate (CO_3_^2−^) ions, suggesting CO_3_^2−^ substitution within the apatite lattice to form carbonated HAp.^[^ ^44^ ^]^

Thermogravimetric analysis (TGA) was also performed on mineralized keratin films to assess their thermal stability as part of mineralization kinetics evaluation. The TGA profiles (FigureS1,S9C, Supporting Information) exhibited a multi‐stage weight loss, where an initial slight weight loss (≈5–10%) was observed between room temperature and ≈150 °C, corresponding to the evaporation of physically adsorbed water molecules. A prominent weight reduction (≈50–60%) occurred between ≈200 and 500 °C, attributable to the thermal decomposition of keratin proteins, primarily due to peptide bond cleavage, disulfide bridge disruption, and degradation of other organic components. The decomposition onset ≈200 °C and completion by 500 °C were consistent across all samples, reflecting keratin's characteristic degradation profile under thermal conditions. Beyond 500 °C, the TGA profiles demonstrated thermal stability up to ≈600 °C, followed by a minor weight loss between 600 and 650 °C, likely corresponding to the decomposition of residual organic constituents not fully degraded in the earlier phase. A stable plateau in weight percent from ≈650 to 800 °C was observed across samples, indicating the presence of inorganic mineral phases. The final residual mass represents the mineral content within the samples and varies according to mineralization duration, increasing progressively from Day 1 to 30, signifying time‐dependent apatite formation within the keratin matrix. This progression demonstrates that the keratin films effectively act as an organic scaffold for mineral deposition, leading to the formation of thermally stable apatite phases over time.

Additionally, Phosphorus‐31 (^31^P) MAS‐NMR spectra displayed a sharp peak at ≈3 ppm, consistent with PO_4_^3−^ groups characteristic of apatite formation in the keratin films at different mineralization time points (Figure S10A, Supporting Information). Mechanical testing of the keratin films further confirmed mineralization effects, with the Young's modulus increasing from 5.1 ± 3.0 GPa to 8.1 ± 3.9 GPa and nano‐hardness from 0.3 ± 0.2 GPa to 0.6 ± 0.3 GPa post mineralization (Figure S10B, Supporting Information).

### 2.7 Keratin Structural Tunability Pre‐ and Post‐Mineralization

Keratin secondary structures were quantified pre‐ and post‐assembly to evaluate their role in directing mineralized motif organization within films (Figure S11 and Table S3, Supporting Information). Increasing keratin concentration at neutral pH (pH 7) enhanced the formation and organization of organic spherulites, with higher concentrations (Ker_5_, Ker_10_) displaying distinct Maltese cross patterns, indicative of crystalline alignment. At low concentrations (Ker_3_), films exhibited a lower random coil (disordered): β‐sheet ratio (ordered) (0.27 ± 0.05) with poorly organized spherulites. In contrast, higher concentrations showed increased ratios (0.68–0.83) prior to drying, which shifted toward β‐sheet dominance upon dehydration, correlating with more organized spherulitic structures and underscoring keratin's conformational versatility in directing biomineralization. Similarly, TEGDMA‐crosslinked keratin films demonstrated concentration‐dependent increases in the random coil: β‐sheet ratio before self‐assembly, followed by increased β‐sheet content and improved spherulite organization after drying. The β‐sheet conformation in proteins is known to facilitate selective stereochemical interactions with crystal faces during biomineralization.^[^ ^41^ ^]^ The transition from disordered random coils to structured β‐sheets, modulated by protein concentration and pH, augments keratin's calcium‐binding capacity and supports the development of organized organic matrices that can act as effective mineralization scaffolds.^[^ ^9^ ^]^

Post‐mineralization analyses revealed that films with a lower random coil: β‐sheet ratio (0.10–0.15) promoted the formation of aligned nanocrystalline apatite within spherulites, while higher ratios (≈0.18 or above) correlated with less organized mineralization (Table S4, Supporting Information). pH also played a critical role; at pH 11, self‐crosslinked films formed mineralized spherulitic structures lacking defined crystalline rods, whereas TEGDMA‐crosslinked films exhibited disorganized mineral aggregates with higher disorder ratios (≈0.33), indicating reduced scaffold‐directed mineral growth.

Based on the previous findings, it is suggested that the keratin mineralization process is multi‐factorial, including protein concentration, pH, and crosslinking, with secondary structure transitions toward β‐sheets enabling the formation of ordered organic spherulites that guide mineral nucleation and alignment. By tuning these parameters, keratin matrices can be engineered to provide supramolecular control over mineral growth, offering a strategy to design biomimetic scaffolds with hierarchical organization for regenerative applications.

### 2.8 Keratin Conformational Changes After Mineralization

**Figure 4:**
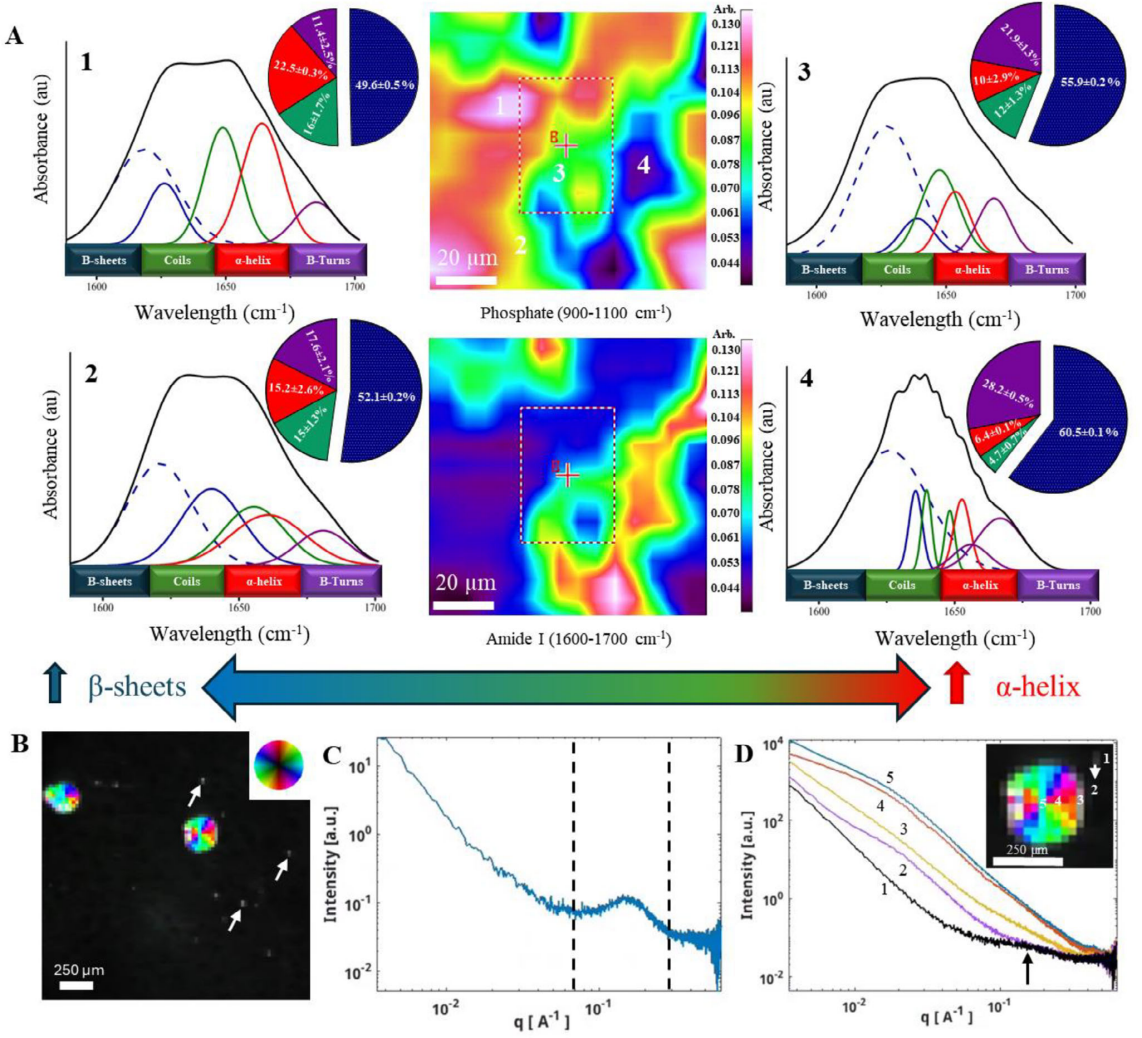
Organic–Inorganic films conformational characterization. A) FTIR imaging of a mineralized film; the heat map at the top represents the distribution of the PO_4_^3>−^ vibrations band region (900–1100 cm^−1^), and amide I band region of the protein (1600–1700 cm^−1^) are represented at the bottom, spectra were collected from four different regions on the heat map (labeled 1, 2, 3, and 4) of a mineralized keratin film according to their proximity to the protein/mineral, corresponding secondary structure deconvolution are presented in the relative pie charts. B) Combined scanning SAXS figure of the mineralized keratin film displaying the integrated intensity of SAXS in the analyzed q‐range of 0.007–0.083 Å^−1^. The brightness of each pixel is given by the intensity of the scattering, the saturation is given by the degree of orientation, and the hue is given by the direction of the strongest scattering. C) Representative SAXS curve from keratin film showing (peak marked with dashed lines) which can be associated with a coil‐like structure, and D) SAXS curves from various positions within the mineralized keratin film as indicated in the inset.

FTIR imaging was performed to investigate keratin's secondary structure changes post‐mineralization. Spectra from regions across the mineralized film (**Figure** **4**A) revealed that β‐sheets dominated near the protein‐rich areas, while α‐helix content increased progressively toward mineralized zones, indicating a conformational shift linked to mineral proximity. This β‐sheet to α‐helix transition, confirmed by ATR‐FTIR deconvolution, suggests β‐sheets promote mineral nucleation and growth, while the emergence of α‐helices near mineralized regions supports apatite formation.

To further assess structural orientation, small‐angle x‐ray scattering (SAXS) analysis was conducted. Mineralized films revealed dispersed mineralized spherulites of ≈250 µm grown on a fibrous keratin substrate with radial crystalline orientation (average orientation ≈0.3) (Figure 4B; Figure S12A–C, Supporting Information) and smaller (≈20 µm) mineralized spherulites lacking preferential orientation (Figure 4B; arrows). SAXS curves showed distinct keratin‐related peaks before mineralization within the q‐range of 0.1–0.25 Å^−1^ (Figure 4C), which is associated with the scattering of a keratin coil‐like structure (protofilament, protofibril, up to a full intermediate filament). While post‐mineralization spectra exhibited additional peaks at a slightly higher q‐range (0.01–0.05 Å^−1^) (Figure 4D, scattering curve 2) compared to the large spherulites (Figure 4D, scattering curves 4, 5), corresponding to mineralized structures (Figure 4C,D). Simultaneous Wide‐angle x‐ray scattering (WAXS) measurements revealed the presence of characteristic peaks of HAp in these mineralized regions (Figure S12D, Supporting Information).

SAXS analysis corroborates FTIR, indicating that larger mineralized spherulites with radial direction correlate with increased α‐helix content, linking keratin dynamics to apatite nucleation. Smaller, unoriented spherulites may signify restricted α‐helix production, underscoring the structural prerequisites for mineral organization. The WAXS validation of HAp in these mineralized areas further substantiates the concept that the structural rearrangement of keratin is pivotal to apatite production.^[^ ^45^ ^]^

### 2.9 Induced Enamel Lesion Surface Characterization In Vitro

**Figure 5:**
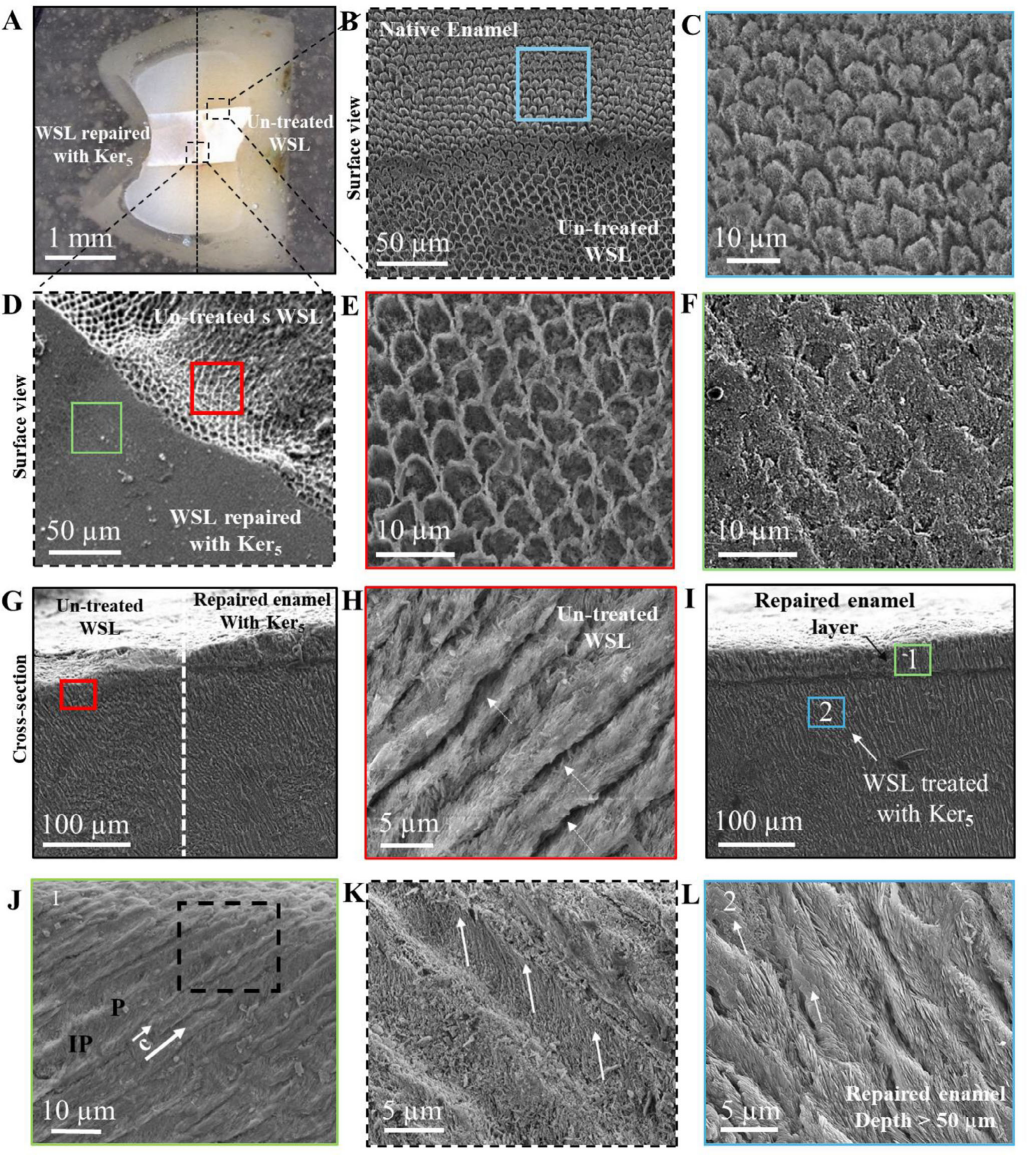
Structural characterization of Keratin treated WSL incubated in mineralization solution. A) Image of a premolar tooth showing the treatment window on its facial surface; WSL on the right has a chalky white characteristic appearance of the WSL, and the keratin‐treated lesion on the left has decreased opacity, demonstrating the remineralization effect of the keratin. B) Interface between healthy and defective enamel demonstrating the lost prism/inter‐prism continua and pores created inside prisms in the WSL zone compared to native enamel. C) High magnification of B showing the native enamel surface. D) Interface between WSL and lesion treated with keratin, where structural differences between the two zones could be noticed. and cross‐sectional. E) High magnification of the untreated WSL surface. F) Surface view of the keratin‐treated lesion showing the newly grown prismatic enamel that has filled the pores. G) Cross section of a WSL window; demonstrating the interface between the newly formed enamel repaired with keratin (right) and the lost prisms in the WSL (left) H) Cross‐sectional view of the enamel pores created due to inducing WSL at a depth between 20 and 50 µm away from the enamel surface where the lost prisms could be depicted, dotted white arrows demonstrate the site of the pores. I) Cross‐sectional view of the WSL treated with keratin demonstrating the reconstructed layer of the newly formed enamel layer with keratin (40–50 µm thick) on top of the lesion; 1 Shows the repaired enamel on top of the WSL treated with keratin at the surface, 2 Shows the repaired enamel at a depth between 50 and 80 µm away from the enamel surface of the WSL. J) High‐magnification of the repaired WSL at the surface after keratin treatment showing the newly formed crystals between the native enamel filling the gaps that was previously induced and demonstrating that the keratin has infiltrated within this gaps and motivated crystal growth. K) High magnification SEM of J showing the keratin‐infiltrated WSL, where some crystals appear to have the same orientation of the prisms along the c‐axis, and others are oriented perpendicularly. L) Reconstructed enamel with keratin filling up the pores from an area ≈50–80 µm away from the enamel surface, with some areas appearing to be filled with organic matrix (White arrows). P: prism, IP: inter‐prism.

An in vitro trial (Illustrated in Figure S13, Supporting Information) using induced enamel WSL models was conducted to explore the potential of keratin to treat artificially induced enamel pores and restore their mechanical properties. WSL induction was achieved after seven days of demineralization. SEM analysis confirmed the successful induction of WSLs, revealing microstructural changes. Intact, polished enamel exhibited densely packed HAp crystals with the characteristic “key‐hole” prism structure (**Figure** **5**A–C). Following WSL induction, a significant loss of crystallites was evident, with visible gaps between enamel prisms (Figure 5D,E; Figure S14A, Supporting Information). Light microscopy further validated WSL formation, demonstrating a chalky white appearance on the lesion surface and in cross‐section compared to adjacent sound enamel (Figure S14B,C, Supporting Information). Optical coherence tomography (OCT) revealed a distinct bright band accompanied by an intensified underlying signal, indicative of increased porosity and light scattering within the lesion body (Figure S14D, Supporting Information).

### 2.10 Keratin‐Assisted Remineralization of WSLs

After treating the WSLs with keratin, improvements in the lesion color and density were detected visually and by light microscopy and OCT grayscale pixel analysis across all conditions. OCT grayscale pixel analysis (Figure S15A, Supporting Information) revealed a decreased value after treatment, indicating increased lesion density. For keratin‐treated WSLs in artificial saliva, grayscale values reduced from 157.81 ± 31.85 to 143.8 ± 45.8, and with resin from 153.16 ± 34.85 to 131.14 ± 43.83 (Figure S15B–D, Supporting Information). In mineralization solution, keratin‐treated WSLs showed reductions from 117.56 ± 49.16 to 88.8 ± 58, and resin‐treated WSLs from 135.52 ± 28.95 to 105.29 ± 44.64 (Figure S15E–G, Supporting Information). Light microscopy grayscale pixel analysis confirmed these trends (Figure S16A, Supporting Information). In UPW, keratin‐treated WSLs showed reductions in pixel values consistent with increased lesion density, comparable to resin‐treated and intact enamel controls. Similar reductions were observed in artificial saliva and mineralization solution, confirming keratin's effectiveness in enhancing WSL density post‐treatment (Figure S16B–D, Supporting Information).

SEM analysis further validated the remineralization potential of keratin incubated in mineralization solution. Surface imaging of treated lesions revealed signs of enamel reconstruction, with clear integration between HAp crystals and keratin (Figure 5F; Figure S17A, Supporting Information). A newly formed enamel‐like layer (≈40–50 µm) was observed on the surface (Figure 5G). Pores and subsurface gaps extending up to ≈50 µm deep, which were previously evident in WSLs (Figure 5H), were repaired throughout the lesion depth post‐treatment (Figure 5I‐L). In contrast, WSLs left untreated and incubated in mineralization solution developed disorganized mineral precipitates lacking prism alignment (Figure S17B,C, Supporting Information). Lesions treated with keratin and incubated in artificial saliva exhibited a similar degree of repair and gap filling as those treated in mineralization solution (Figure S18, Supporting Information). In comparison, resin‐infiltrated WSLs demonstrated resin filling enamel gaps and forming a surface layer; enamel prisms above the resin interface appeared as clumped structures lacking defined crystal organization (Figure S19, Supporting Information).

When keratin‐treated WSL were incubated in UPW, they infiltrated the WSL and formed a coating on the surface with no evidence of mineral repair. SEM cross‐sectional imaging revealed keratin infiltration into lesion pores and formation of a homogeneous ≈10 µm surface layer (Figure S20A,B, Supporting Information). EDX analysis confirmed this layer's high carbon and sulfur content, with the spectral intensity increasing progressively as the analysis moves from native enamel to the keratin‐coated area. (Figure S20C–F, Supporting Information), while enamel exhibited high calcium and phosphorus (Figure S20G,H, Supporting Information). Point analysis further confirmed the presence of sulfur and absence of calcium and phosphorus in the keratin layer, opposite to the enamel side (Figure S20I,J, Supporting Information). These results indicate that keratin forms a stable organic scaffold on demineralized enamel, likely facilitating mineral nucleation upon subsequent mineralization.

**Figure 6:**
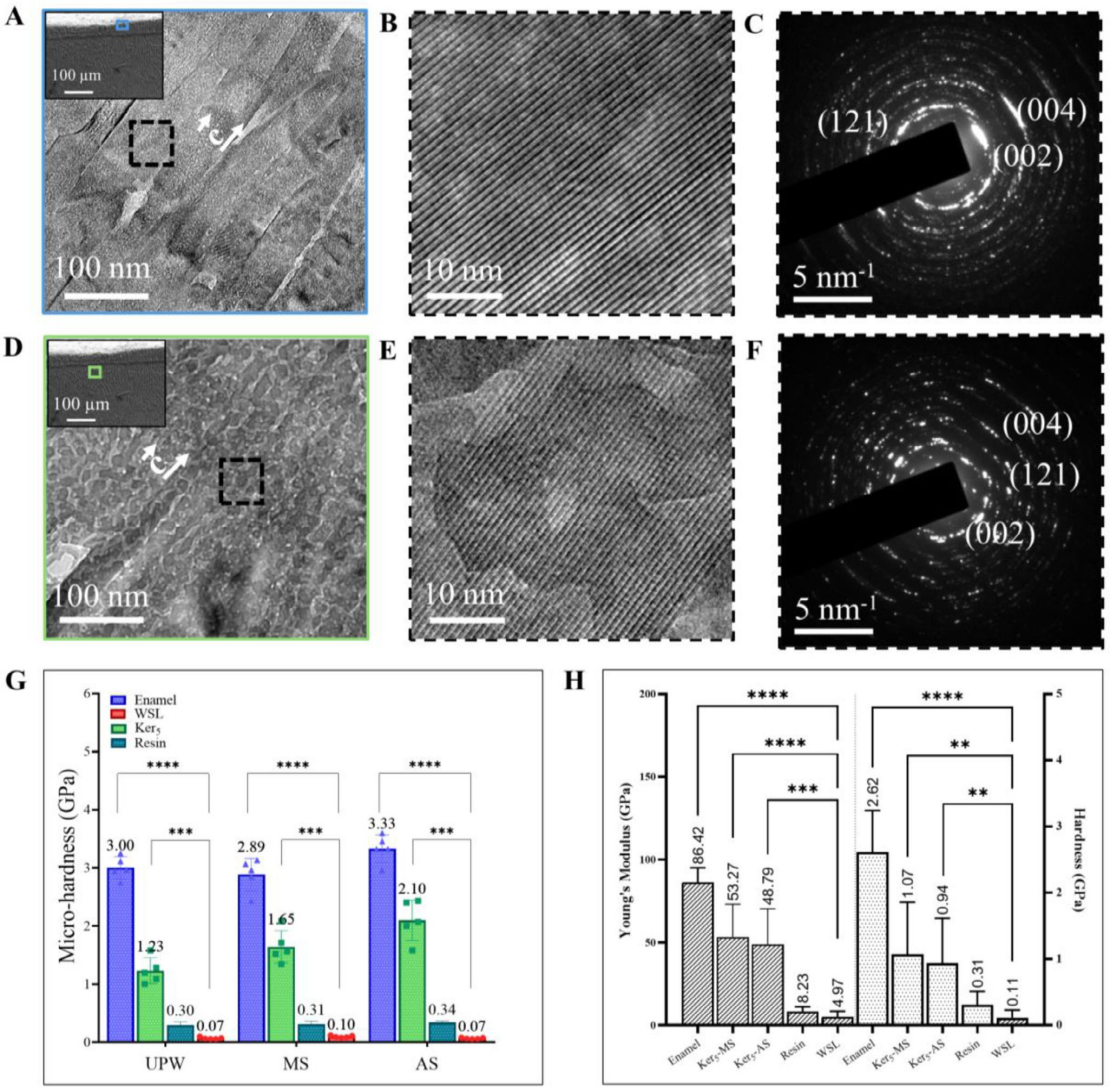
Crystallographic and Mechanical Characterization of Keratin treated WSLs. A) HR‐TEM of FIB lamella milled from an area close to the surface of a WSL treated with Ker_5_ showing reconstructed enamel prisms, ^*^Inset showing zone where the lift‐out was milled. B) High magnification of the outlined square in A. C) SAED demonstrating the crystal diffraction pattern of the repaired region at the surface. D) HR‐TEM of FIB lamella milled from an area ≈ 60 µm away from the surface of a WSL treated with Ker_5_, demonstrating reconstructed enamel prisms demonstrating a mosaic pattern. ^*^ Inset showing zone where the lift‐out was milled. E) High magnification of the outlined square in D. F) SAED demonstrating a continuous crystal diffraction pattern at the bulk of the repaired lesion, as well as diffraction comparable to apatite crystals. G) Knoop microhardness analysis (Mean ± S.D.) of the WSLs surface before and after treatments (n=5). The WSLs treated with keratin and incubated in either UPW, MS or AS, showed significant improvements in the microhardness (GPa) of the repaired enamel surface compared to WSL before treatment and to those treated with resin infiltrant. * indicates significant difference, where significance at p <0.001. H) Surface nanoindentation measurements (Mean ± S.D.) of the WSLs surface before and after treatments (n=3). The WSLs treated with keratin and incubated in either MS or AS, revealed enhanced elastic modulus and hardness of the repaired enamel surface compared to WSL before treatment and to those treated with resin infiltrant. “*” indicates significant difference, where significance at p <0.01. MS: mineralization solution, AS: artificial saliva.

HR‐TEM of FIB‐milled lamellae from keratin‐treated lesions captured near the surface and ≈60 µm into the bulk revealed bundles of newly formed nanocrystals aligned with native enamel crystals along the c‐axis, showing continuous integration within prisms and confirmed apatite phase by SAED (**Figure** 6  A‐F). A mosaic pattern in deeper crystals suggested variation in crystallographic orientation, demonstrating keratin's capacity to repair enamel across lesion depth.

### 2.11 Keratin‐Treated WSLs Conformation and Mechanical Properties

FTIR spectroscopy was employed to investigate keratin‐enamel interactions and their role in HAp nucleation during remineralization (Figure S21, Supporting Information). Native enamel exhibited no detectable protein bands due to its highly mineralized surface, while WSLs showed distinct secondary structures with high β‐turn content (49.33 ± 0.19%), β‐sheets (25 ± 0.6%), and α‐helices (15.49 ± 0.62%). This indicated superficial crystal dissolution, exposing intrinsic enamel proteins. WSLs treated with keratin (without mineralization) demonstrated a substantial increase in β‐sheets (54.74 ± 0.33%) and a decrease in β‐turns (22.80 ± 1.66%), with stable α‐helices (15.13 ± 0.11%). Upon incubation in mineralization solution, keratin‐treated lesions showed an increase in β‐turns (52.95 ± 1.84%) and α‐helices (23.15 ± 1.95%), with a reduction in β‐sheets (16.67 ± 3.1%), indicating structural rearrangements driven by keratin‐mineral interactions and variable protein‐to‐mineral ratios within the repaired enamel. These findings suggest an interaction between keratin and the minerals provided by the mineralization solution, leading to secondary structural rearrangements. The increased standard deviation observed in the secondary structure content of keratin‐treated WSLs may be attributed to some variations in the protein‐to‐mineral ratio within the repaired enamel.

Knoop microhardness testing (Figure 6G) showed keratin‐treated lesions in UPW achieved 1.23 ± 0.22 GPa, compared to 3.00 ± 0.19 GPa in healthy enamel and 0.07 ± 0.02 GPa in untreated WSLs. In mineralization solution, keratin‐treated lesions reached 1.65 ± 0.30 GPa, while in artificial saliva, microhardness increased to 2.10 ± 0.35 GPa, significantly higher than resin‐infiltrated samples under similar conditions (0.31–0.34 GPa). Nanoindentation measurements further validated the functional restoration of keratin‐treated WSLs (Figure 6H). Pre‐lesion enamel exhibited an elastic modulus of 86.42 ± 8.67 GPa and hardness of 2.62 ± 0.67 GPa, which significantly decreased after WSL induction (4.97 ± 3.45 GPa and 0.11 ± 0.12 GPa, respectively). Post‐keratin treatment, the modulus and hardness improved to 53.27 ± 19.78 GPa and 1.07 ± 0.78 GPa, respectively, in lesions incubated in mineralization solution, with similar improvements observed in artificial saliva (48.14 ± 21.16 GPa and 0.94 ± 0.68 GPa). In contrast, resin‐treated lesions showed modest improvements (8.23 ± 2.95 GPa and 0.31 ± 0.20 GPa). Bulk nanoindentation analysis (Figure S22, Supporting Information) confirmed these trends, with intact enamel showing a modulus of 80.11 ± 18.31 GPa and hardness of 3.26 ± 0.91 GPa, which decreased by ≈91–95% post‐WSL induction. Following keratin treatment, substantial recovery was observed, with mineralized WSLs in mineralization solution exhibiting a modulus of 54.83 ± 19.69 GPa and hardness of 1.15 ± 0.56 GPa, and keratin‐treated lesions in artificial saliva demonstrating similar restoration (55.56 ± 12.95 GPa and 1.01 ± 0.38 GPa).  

Collectively, these results demonstrate that keratin treatment not only modulates secondary protein structures to promote mineral nucleation but also restores enamel mechanical properties both at the surface and within the lesion depth, supporting its potential as a functional biomimetic strategy for WSL repair.

## 3 Conclusion

This study establishes a pre‐clinical framework for using water‐based keratin platforms to repair enamel demineralization lesions, demonstrating keratin's potential as a cheap, abundant, and biocompatible biomaterial for functional enamel regeneration. Keratin films self‐assembled into β‐sheet‐rich spherulitic architectures, forming organized nucleation sites that directed the growth of enamel‐like mineral layers with aligned apatite nanocrystals and fluoride incorporation. The transition from β‐sheets to α‐helix and β‐turn structures upon mineralization underscores keratin's dynamic role in orchestrating hierarchical mineralization, mimicking natural enamel formation. These newly formed crystals exhibited significant recovery in hardness and elastic modulus, restoring both surface and subsurface mechanical integrity beyond that achievable with resin infiltration, while preserving crystalline architecture. Importantly, keratin facilitated controlled mineral phase development, transitioning ACP to organized apatite, confirming its capacity to mediate biomineralization efficiently.

Collectively, these findings establish keratin as a clinically viable, sustainable biomaterial for enamel repair, enabling functional regeneration of enamel architecture with a simple, solvent‐free fabrication process. Future studies should focus on optimizing keratin's structural tuning and functionalizing it with additional acidic domains to enhance mineral binding affinity, while conducting systematic in vitro and in vivo cellular studies to evaluate cytocompatibility, bioactivity, and integration within hard tissue environments, thereby supporting its broader application in dental tissue engineering and regenerative medicine. Beyond enamel repair, keratin‐based matrices hold promise for addressing bony defects, dentine hypersensitivity, and erosive tooth wear, with broad implications for dental and biomedical fields. The simplicity, scalability, and affordability of this system position keratin as a resourceful platform for advancing sustainable, clinically feasible regenerative strategies in tissue engineering and structural biomimetics.

## 4 Experimental Section

### Materials

All materials used in this study were purchased from Sigma–Aldrich (Gillingham, UK), unless specified otherwise. Chemicals were used as received without any further processing.

### Keratin Self‐assembly and Film Fabrication

Films were fabricated by dissolving lyophilized keratin powder (30–100 mg mL^−1^) in Milli‐Q water at room temperature at concentrations of 3, 5, and 10 w/v % and were named ker_3_, ker_5_, and Ker_10_, respectively. For some films, the resultant mixture was crosslinked to the cysteine residues in the Keratin using Triethylene glycol dimethacrylate (TEGDMA). TEGDMA concentrations were 0.04 w/v%, (TE_0.5_), 0.4 w/v % (TE_1_) and 0.8 w/v% (TE_2_). Mixtures were then drop‐casted on top of polydimethylsiloxane and left to dry overnight to induce self‐assembly; the resulting films were washed in deionized water for 1 day. Secondary structure conformation was done using Fourier transform infrared (FTIR) spectroscopy imaging, and morphological analyses were carried out using light microscopy, atomic force microscope (AFM), and scanning electron microscopy (SEM).

### Films Mineralization

Mineralization of the keratin films was induced by adding 2 mM HAp powder and 2 mM of sodium fluoride in deionized water with continuous stirring. To dissolve the powder completely, 69% nitric acid was added dropwise into the solution slowly until the powder was completely dissolved. Ammonium hydroxide solution (30%) was added dropwise until the pH reached 6.^[^ ^46^ ^]^ The keratin films were incubated in 50 mL of the HAp solution at 37 °C. The films were then thoroughly rinsed with UPW, sonicated for 2 min in a water bath to remove any precipitates, and stored at room temperature until further analysis.

### Attenuated Total Reflection‐Fourier Transform Infrared (ATR‐FTIR) Spectroscopy

ATR‐FTIR analysis was conducted to investigate the secondary structures of the keratin solutions and films using a Spectrum One FTIR Spectrometer (PerkinElmer, Buckinghamshire, UK) in ATR mode, using a white light source and an InGaAs detector. Spectra were taken between wavenumber 4000–700 cm^−1^ by averaging 32 scans per sample at a resolution of 2 cm^−1^. The amide I spectral region (1700–1600 cm^−1^) was analyzed to compare the keratin secondary structure composition using OriginPro 8.5 software (Microcal Inc.). The assignments of spectral bands were as follows: 1610–1627 cm^−1^ intermolecular β‐sheets, 1628–1642 cm^−1^ β‐sheets, 1643–1650 cm^−1^ random coils, 1650–1659 cm^−1^ α‐helix, and 1660–1699 cm^−1^ β‐sheets/β‐turns.^[^ ^47^ ^]^

### Scanning Electron Microscopy (SEM)

Samples were mounted on aluminum stubs via carbon‐adhesive tape and were coated with 10 nm thick gold coating using an auto sputter coating machine coating (Leica EM ACE600 sputter coater, Milton Keynes, UK). SEM images were obtained using a Schottky field‐emission JSM‐7610F SEM (JEOL Ltd., Hertfordshire UK) operated at 10 kV, with the surface topography being observed using the secondary electron detector. Images were then transferred to ImageJ (NIH, US) for quantitative analysis. Induced enamel lesions were prepared by etching with phosphoric acid 35% for 20 s and rinsed for 20 s to remove the smear layer.

### High‐Resolution Transmission Electron Microscopy (HR‐TEM)

The FIB‐prepared lamellae were characterized using a Thermo Fisher 60–300 kV Spectra Ultra TEM equipped with an Ultra‐X EDS detector and Cs aberration corrector. Images were acquired on a 4k × 4k Ceta‐S detector. Data was post‐processed using Velox software, version 3.8. The obtained images were analyzed using the Gatan Microscopy Suite (GMS 3) software. For the analysis of crystal phases present in the samples, d‐values obtained from SAED patterns were compared against the PDF2 database (ICDD, USA, release 2009).

### Enamel Sections Preparation

Extracted human non‐carious molar teeth (with HRA approval from NHS Research Ethics Committee, reference number: 16/SW/0220) were collected and an informed written consent from all participants was obtained prior to the research. White spot lesions (WSLs) were created on the enamel surface Teeth were examined under a white light microscope (GXM‐XPLPOLTEC‐5, UK) to make sure no caries or cavities were present. The facial surface of the samples was then placed facedown in a silicone mold and was then embedded into clear acrylic resin (Oracryl, Bracon, UK).

Enamel sections were obtained using a water‐cooled rotary diamond saw (XL 12205, Benetec Ltd., UK). Sections were carefully polished using a polishing machine (MetaServ 3000, Buehler, USA) with the aid of silicon carbide grinding papers (Struers, UK) from coarse to fine as follows (P500, P1200, P2500, and P4000). The polishing direction was altered by 90 degrees, and ultra‐sonification was carried out after each step. Following polishing, all samples were stored in distilled water before further treatment. Samples were analyzed using light microscopy, Optical coherence tomography (OCT), SEM, and HR‐TEM before and after treatment. Mechanical properties were also quantified using Microhardness and nanoindentation testing.

### Statistical Analysis

All data are reported as mean ± SD using GraphPad Prism ver. 10.2 (GraphPad Software, USA). The Shapiro–Wilk test was used to assess normality, guiding the use of parametric or nonparametric tests. For comparisons between two groups, two‐sided Student's unpaired *t*‐tests were used. For comparisons among multiple groups, one‐way ANOVA with Tukey's HSD post‐hoc test was applied, with alpha adjustment for multiple comparisons. The level of statistical significance was set as indicated in the figure legends for each analysis.

## Conflict of Interest

The authors declare they have no competing interests.

## Author Contributions

S.G. and S.E. conceived the project and designed the experiments. S.G. carried out experiments. S.E. supervised the study. S.G., A.P., and E.R. extracted the Keratin. A.P. conducted and analyzed FTIR on extracted Keratin. S.G. and E.R. fabricated the keratin films used in this study, performed crosslinking studies, and carried out the DLS studies. S.G. and E.R. conducted and analyzed FTIR data on keratin films. S.G. and K.L.A.C. conducted FTIR imaging data. S.G., E.R., S.E., D.A., L.A., and R.F. performed SEM imaging on the keratin films. S.G. and S.E. conducted polarized light microscopy experiments. S.G., S.E., R.L.C., D.A., and K.C. planned, conducted, and analyzed AFM data, FIB‐SEM lift‐out, TEM images, and SAED patterns on the keratin films. S.G. collected and analyzed the NMR data. S.G., S.E., D.A., M.L., and M.C. planned, conducted, and analyzed SAXS data. S.E., S.G., and A.B. analyzed and interpreted the in vitro mineralization model. S.K., S.H., and S.G. made the WSL models. S.H., S.G., and S.H. performed microhardness testing. G.D.S., S.G., S.E., and N.M.P. planned, conducted, and interpreted the nano‐indentation experiments. S.G. and E.W. performed the FIB‐SEM and TEM experiments on the tooth samples. S.G. analyzed TEM images and SAED patterns on teeth samples. S.G. and S.E. interpreted the data and wrote the manuscript. All authors discussed the results and commented on the manuscript.

## Supporting information



Supporting Information

## Data Availability

The data that support the findings of this study are available from the corresponding author upon reasonable request.
