# Supplementary material for: Biomimetic Mineralization of Keratin Scaffolds for Enamel Regeneration
Source: Adv Healthc Mater. 2025 Aug 12;14(30):e02465. doi: 10.1002/adhm.202502465 (PMC12645081; doi:10.1002/adhm.202502465)
Supplement: Supplementary file 1 — Supporting Information [file ADHM-14-0-s001.pdf]

ADVANCED HEALTHCARE MATERIALS

Supporting Information

for *Adv. Healthcare Mater.*, DOI 10.1002/adhm.202502465

Biomimetic Mineralization of Keratin Scaffolds for Enamel Regeneration

*Sara Gamea, Elham Radvar, Dimitra Athanasiadou, Ryan Lee Chan, Giacomo De Sero, Ecaterina Ware, Sunie Kundi, Avir Patel, Shwan Horamee, Shuaib Hadadi, Mads Carlsen, Leanne Allison, Roland Fleck, Ka Lung Andrew Chan, Avijit Banerjee, Nicola Pugno, Marianne Liebi, Paul T Sharpe, Karina Carneiro and Sherif Elsharkawy**

Supporting Information for

Biomimetic Mineralization of Keratin Scaffolds for Enamel Regeneration

Sara Gamea[1,2], Elham Radvar[1], Dimitra Athanasiadou[3,4], Ryan Lee Chan[5], Giacomo De Sero[6], Ecaterina Ware[7], Sunie Kundi[1], Avir Patel[8], Shwan Hormaee[1], Shuaib Hadadi[1], Mads Carlsen[9], Leanne Allison[10], Roland Fleck[10], Ka Lung Andrew Chan[11], Avijit Banerjee[1], Nicola Pugno[6,12], Marianne Liebi[4,9,13], Paul T Sharpe[14], Karina Carneiro[3,5], Sherif Elsharkawy*[1,15]

1. *Centre for Oral, Clinical, and Translational Sciences, Faculty of Dentistry, Oral & Craniofacial Sciences, King's College London, London, SE1 9RT, United Kingdom*
2. *Tanta University, Faculty of Dentistry, Department of Restorative Dentistry, Tanta, 31111, Egypt*
3. *Faculty of Dentistry, University of Toronto, Toronto, ON M5G 1G6, Canada*
4. *Department of Physics, Chalmers University of Technology, 412 96 Gothenburg, Sweden*
5. *Institute of Biomedical Engineering, University of Toronto, Toronto, ON M5S 3G9, Canada*
6. *Laboratory for Bioinspired, Bionic, Nano, Meta, Materials & Mechanics, Department of Civil, Environmental and Mechanical Engineering, University of Trento, 38123 Trento, Italy*
7. *Department of Materials, Royal School of Mines, Imperial College London, London, SW7 2AZ, United Kingdom*
8. *Department of Chemistry, King's College London, London, SE1 1DB, United Kingdom*
9. *Photon Science Division, Paul Scherrer Institute, 5232 Villigen PSI, Switzerland*
10. *Centre for Ultrastructural Imaging, King's College London, New Hunts House, Guys Campus, London SE1 1UL, UK.*
11. *Institute of Pharmaceutical Science, King's College London, SE1 9NH, UK*
12. *School of Engineering and Materials Science, Queen Mary University of London, Mile End Road, London E1 4NS, UK*
13. *Institute of Materials, Ecole Polytechnique Fédérale de Lausanne (EPFL), 1015 Lausanne, Switzerland*
14. *Centre for Craniofacial and Regenerative Biology, Faculty of Dentistry, Oral & Craniofacial Sciences, King's College London, London, SE1 9RT, United Kingdom*
15. *Prosthodontics Department, Dental Directorate, Guy's and St Thomas' NHS Trust, London, SE1 9RT, United Kingdom*

***Correspondence:** Dr. Sherif Elsharkawy,

Email: sherif.elsharkawy@kcl.ac.uk

SUPPLEMENTARY MATERIALS AND METHODS

Extraction of Sheep Wool Keratin

The Keratin was successfully extracted from sheep's wool (Ovis aries). Wool was thoroughly rinsed with deionized water, dried, and defatted by Soxhlet extraction for 6 hours using hexane and dichloromethane 1:1 v/v for refluxing. Cleaned wool (10 g) was mixed with 7M urea (1.26 mol, 180 mL), Sodium Dodecyl Sulfate (0.021 mol, 6 g) and 2-mercaptoethanol (0.21 mol, 15 mL) in a 500 mL round-bottom flask. The mixture was heated at 50°C for 48 hours with continuous stirring and maintained in a neutral pH range. The resultant mixture was filtered through a 120 stainless-steel mesh sieve and then centrifuged for 30 mins at 6,000 rpm, and the supernatant was subsequently dialyzed (5 kDa cut off) against deionized water (3.5 L) for three days until a colorless clear solution was obtained with regular changing of the outer water (two to three times). Aliquots of the reduced keratin solution were kept in a freezer at -80°C for 4 hours and then freeze-dried (VirTis SP Scientific Sentry 2.0, Ipswich, UK) until a fine lyophilized white interwoven fibrous powder remained.

Bicinchoninic Acid (BCA) Assay.

Following protein extraction, the resultant protein extract concentration was determined using BCA. A series of dilutions of known concentrations were prepared from the freeze-dried keratin and assayed alongside the reference protein, which is bovine serum albumin. A linear curve at 562 nm absorbance was reported for the keratin concentrations and were confirmed based on the standard curve of Bovine Serum Albumin A linear curve at 562 nm absorbance was reported for the keratin concentrations and were confirmed based on the standard curve of Bovine Serum Albumin.

Sodium Dodecyl Sulphate-Polyacrylamide Gel Electrophoresis Analysis

To determine the molecular weight of the resultant extracted solution, lyophilized keratin powder from two different batches was analyzed using SDS-PAGE. Lyophilized Keratin of 2 different keratin concentrations (8 and 4 w/v%) for two different batches were mixed each with aliquots of LDS sample buffer (5 µL) and TCEP (0.5 µL) and heated at 70°C for 10 minutes. 10 µL of each solution were then loaded on 12% Bis-Tris NuPAGE® precast polyacrylamide gel (ThermoFisher

Scientific, UK). Electrophoresis was performed at 200 V, 125mA for 40 min. The gels were then dyed by Coomassie Brilliant Blue for 4 h and destained overnight with deionized water under constant shaking.

Liquid Chromatography with Tandem Mass Spectrometry (LC-MS/MS) Analysis.

In-gel reduction, alkylation and digestion with trypsin were performed on the keratin SDS-PAGE gel band samples after excision and prior to subsequent analysis by mass spectrometry. Cysteine residues were reduced with dithiothreitol and derivatized by treatment with iodoacetamide to form stable carbamidomethyl derivatives. Trypsin digestion was carried out overnight at room temperature after initial incubation at $37^{o}C$ for 2 hours. The peptide sample was then resuspended in 30 ml of resuspension buffer (2% v/v ACN in 0.05% formic acid), 10 ml of which was injected to be analyzed by LC-MS/MS. Chromatographic separation was performed using a U3000 UHPLC NanoLC system (ThermoFisherScientific, UK). Peptides were resolved by reverse-phase chromatography on a 75 mm C18 Pepmap column (50 cm length) using a three-step linear gradient of 80% acetonitrile in 0.1% formic acid. The gradient was delivered to elute the peptides at a flow rate of 250 nl/min over 60 min starting at 5% B (0-5 minutes) and increasing solvent to 40% B (5-40 minutes) prior to a wash step at 99% B (40-45 minutes) followed by an equilibration step at 5% B (45-60 minutes). The eluate was ionized by electrospray ionization using an Orbitrap Fusion Lumos operating under Xcalibur v4.1.5 (ThermoFisher Scientific, UK).

The instrument was first programmed to acquire using an Orbitrap-Ion Trap method by defining a 3s cycle time between a full MS scan and MS/MS fragmentation. Orbitrap spectra (FTMS1) were collected at a resolution of 120,000 over a scan range of m/z 375-1500 with an automatic gain control (AGC) setting of 4.0E5 with a maximum injection time of 35 ms. Monoisotopic precursor ions were filtered using the charge state (+2 to +7) with an intensity threshold set between 5.0e3 to 1.0e20 and a dynamic exclusion window of 35s ± 10 ppm. MS2 precursor ions were isolated in the quadrupole set to a mass-width filter of 1.6 m/z. Ion trap fragmentation spectra (ITMS2) were collected with an AGC target setting of 1.0e4 with a maximum injection time of 35 ms with CID collision energy set at 35%. This method takes advantage of multiple analyzers in the Orbitrap Fusion Lumos and

drives the system to use all available parallelizable time, resulting in decreasing dependence on method parameters. Raw mass spectrometry data were processed into peak list files using Proteome Discoverer (ThermoScientific; v2.2). The raw data file was processed and searched using the Mascot search algorithm (v2.6.0); www.matrixscience.com) and the Sequest search algorithm[1] against the UniProt All Taxonomy database (563,552 entries). The database output file was uploaded to Scaffold software® (version 4.11.1; www.proteomesoftware.com) for visualization and manual verification. The data was searched at a stringency threshold of 1% false discovery rate (FDR) for protein with a minimum of one peptide per protein and peptide threshold set to 95% probability as determined by Mascot and Sequest in the Proteome Discoverer method. The data shows proteins with at least 99% identification probability that could be present in our Keratin and their amino acid coverage when compared to the database.

Circular Dichroism (CD) Spectroscopy

The secondary structure of Keratin in aqueous solutions of different pH-values was investigated by CD spectroscopy (Chirascan™ CD Spectrometer, Applied Photophysic Limited, UK) equipped with a temperature controller. The final pH for both solutions were stable over time. The keratin solutions (0.2 mg/ml) were prepared in Milli-Q water. To test the effect of Calcium ions on the keratin secondary structure, lyophilized Keratin (0.2 mg/ml) was dissolved in 5mM Calcium Chloride ($CaCl_2$). A quartz cuvette with a 0.5 mm path length was used for the measurements, and CD spectra were obtained by signal integration three scans from 190 to 260 nm at a scan rate of 50 nm/min with a bandwidth of 1 nm. The spectra were acquired at 25°C. The solution was equilibrated for 5 min before scanning. CD data sets were then deconvoluted using Dichroweb® [2] web server for the calculation of protein secondary structures.

Dynamic Light Scattering (DLS)

In order to optimize the formation of the keratin films, DLS was performed to measure changes in the particle size of Keratin in solution and compare their charges. Zetasizer (Nano-ZS ZEN 3600, Malvern Instruments, UK) was used to measure both the Z-potential and Z-average measurements. The keratin solutions (0.2 mg/ml) were prepared in Milli-Q water at a pH of 25˚C. Also, to test the

interaction of Keratin with Calcium salts, lyophilized keratin (0.2 mg/ml) was dissolved in 5 mM $CaCl_2$. Each sample was equilibrated for 5 min before measurements.

Isothermal Titration Calorimetry (ITC)

The binding interaction between calcium ions and keratin was investigated using ITC on a MicroCal PEAQ-ITC instrument (Malvern Panalytical, UK) at 16°C. Keratin was dissolved in 10 mM Tris-HCl buffer (pH 7.4) to a final concentration of 40 µM and loaded into the sample cell (volume 200 µL). The titrant, 50 mM $CaCl_2$ prepared in the same buffer, was loaded into the syringe (volume 40 µL). A series of 1 µL injections of $CaCl_2$ were administered into the keratin solution with a 150 s interval between injections to ensure complete thermal equilibration. The stirring speed was set at 750 rpm to maintain homogeneity during the titration. Control experiments consisting of titrating $CaCl_2$ into buffer alone were conducted under identical conditions to account for heat of dilution, and these values were subtracted from the experimental data. The raw heat change per injection (µcal/s) was integrated and plotted against the molar ratio of Ca^{2+} to keratin using the MicroCal PEAQ-ITC Analysis Software. Data were fitted to a one-set-of-sites binding model to extract thermodynamic parameters, including binding stoichiometry (N), dissociation constant (K_D), enthalpy change (ΔH), and entropy contribution (–TΔS). All measurements were performed in triplicate to ensure reproducibility.

Quantification Of the Keratin Thiol Groups

Thiol groups in the different keratin films were quantified by using Ellman's assay[3]. Ellman's reagent [5,5'-dithio-bis-(2-nitrobenzoic acid) (DTNB)] (Thermo Scientific®) is a versatile water-soluble compound used for quantitating free sulfhydryl groups in solution. The solution of this compound produces a measurable, yellow-colored product when it reacts with sulfhydryl groups. The general procedure involves the reaction between DTNB and thiol groups from sulfhydryl molecules followed by the release of TNB anion. Sulfhydryl groups were estimated in the keratin sample by comparison to a standard curve composed of known concentrations of a sulfhydryl-containing compound. A set of L-cysteine

standards of known concentrations was prepared in a reaction Buffer of 0.1M sodium phosphate containing 1mM EDTA of pH 8.0.

A set of test tubes were then prepared, each containing 50µL of Ellman's Reagent Solution and 2.5mL of Reaction Buffer. Three keratin samples, Ker_5, Ker_5TE_1, and Ker_5TE_2, were prepared and tested. Triplicates from each keratin sample, as well as standards (250µL), were added to the separate test tubes previously prepared and were properly mixed. All samples were incubated in 96-well plates for 15 minutes at room temperature. Keratin samples' free thiols were quantified from the remaining keratin mixture during the film drying process at 3 different time points: baseline, after 30 minutes and after 1 hour to determine the free thiols. Absorbance was measured at 412 nm using a UV/Vis spectrometer (CLARIOstar® Plus, BMG Labtech, Germany).

Bright-Field Light Microscopy.

Films were observed by cross-polarized light microscopy (GXM-XPLPOLTEC-5, UK) with an air objective 4, 10, 20 and 40x in which the polarizer and analyser were fixed perpendicularly to each other.

Atomic Force Microscopy (AFM)

Keratin films with or without mineralization on glass slides were imaged in tapping mode under ambient conditions using a MultiMode® AFM with a Nanoscope III controller (Digital Instruments, Santa Barbara) and OTESPA-R3® cantilevers (Bruker, California). Image analysis was performed using NanoScope® analysis (Bruker, California), where the images were flattened to remove curvature and slope.

FTIR Imaging

FTIR spectroscopic imaging data were acquired using a Perkin Elmer Spotlight 400 imaging system used at high magnification mode for FTIR mapping. FTIR images were acquired using ATR imaging modes. ATR images were recorded with the Perkin Elmer Spotlight 400 ATR imaging adapter using Germanium crystal. Spectra were collected over wavenumbers between 4000 to 750 cm^{-1}. Spectral measurements were acquired from different regions of the keratin films according to their proximity to the protein and/or mineralized areas with a pixel size of 1.56 µm per pixel for the organic spherulite as we thought that high resolution would be

better to achieve fine details of the spherulites, while spectra were acquired at 6.25 µm per pixel for the mineralized films, at a spectral resolution of 4 cm^{-1}. Background measurements were acquired in a region with no films and 32 scans per pixel. The ATR crystal was gently placed in contact with the films using minimal pressure to ensure good contact. The images were collected using the PerkinElmer Spotlight 400® and were then processed using the SpectrumIMAGE® software.

Magic angle spin- nuclear magnetic resonance (MAS-NMR) for chemical analysis of the mineralized films.

In order to provide detailed information on the atomic structure of keratins in solid form, solid-state Fluorine-19 (^{19}F) and Phosphorus-31 (^{31}P) MAS-NMR analysis were conducted using a 14.1 Tesla spectrometer (600 MHz, Bruker, Coventry, UK) at a Larmor frequency of 564.5 MHz under spinning conditions of 22 kHz in a 2.5mm rotor to investigate the fluoride and phosphorous compounds in the mineralized films over time. All samples were crushed into fine powder using gyro mill machine (Gyro mill, Glen Cresto, London, UK) and then analyzed. The spectra were acquired from a single-pulse experiment of 60 s recycle duration. The ^{19}F chemical shift scale was calibrated using the −120 p.p.m. peak of 1M of NaF solution. H_3PO_4 was the reference material for the chemical shift in ^{31}P. Spectra were acquired for 4 h with accumulation of 240 scans.

Thermogravimetric Analysis (TGA)

TGA was performed to assess the thermal stability of mineralized keratin protein films across six time points (Days 1-30). Approximately 5–7 mg of each film sample was loaded into a platinum pan and analyzed using a thermogravimetric analyzer TGA 5500, (TA Instruments, New Castle, DE, USA). The temperature was ramped from 25 °C to 800 °C at a constant heating rate of 10 °C/min. A dual-atmosphere protocol was employed: the samples were heated from 25 °C to 600 °C under a nitrogen atmosphere (flow rate: 60 mL/min) to enable pyrolysis of the organic matrix, followed by a switch to air (flow rate: 60 mL/min) from 600 °C to 800 °C to facilitate complete combustion of residual organics, leaving only the inorganic mineral content. The weight loss profiles were recorded continuously, and the residual mass at 600°C and 800°C was used to evaluate the thermally stable

mineral content within the films. All measurements were performed in triplicate to ensure reproducibility.

Focused Ion Beam (FIB)- SEM

FIB milling and deposition were performed on the keratin films using a Thermo Scientific™ Helios™ 5 UC DualBeam or Zeiss Crossbeam 350 FIB-SEM at gallium ion beam parameters, 30 kV and 1 nA, except for a final low-voltage cleaning step done with a 2 kV beam to provide additional gentle thinning while reducing Ga implantation and amorphization damage. The region of interest (ROI), approximately 20 x 20 μm^2-wide, was coated with a thin layer of carbon and a thicker, ~2 µm, tungsten layer by electron and ion beam deposition. The specimen was extracted with a micromanipulator and attached to a Cu-grid. Sample pores were filled with tungsten deposition to improve the structural integrity of the sample during thinning. The lamella was thinned with three or four windows, ~3 µm wide, depending on the size of the mineral specimen chosen. For the enamel samples treated with Keratin, FIB-SEM was undertaken using a Thermo Scientific™ Helios™ 5 CX DualBeam, for which the platinum electron beam deposition parameters were set to 5 kV and 0.69 nA, gallium ion beam platinum deposition was set to 30 kV and 0.43 nA, milling the trenches was done at 30 kV and 21 nA, and cleaning cross-section at 30 kV and 25 nA. Electron beam deposition was done at a stage tilt of 0°, ion beam deposition and trench milling were done at 52° (plus a small over-tilt angle of ~0.7° to obtain parallel front and back faces for thinning), and lift-out and attachment were done at 0°. Samples were thinned down to 60-80 nm for electron transparency with gentle polishing at 2 kV and 23 pA.

Scanning Small/Wide-Angle X-Ray Scattering (SAXS/WAXS)

Scanning SAXS scattering experiments were conducted at the cSAXS beamline, Swiss Light Source (SLS), located at the Paul Scherrer Institute (PSI) in Switzerland. A micro-focused X-ray beam was utilized for these experiments, with photon energy set at 12.4 keV using a Si (111) double crystal monochromator. Measurements were made using a beam size of 20 µm on non-mineralized and mineralized keratin films raster scanning an area of 2 x 2 mm. To minimize air scattering and absorption, an evacuated flight tube was positioned between the sample and the detector. The keratin film samples were mounted on a motorized

stage capable of movement in two axes within the plane perpendicular to the incoming beam (x-y plane), enabling raster scanning. Fly-scanning was employed in the vertical direction (y) during the experiments, with a step size of 20 μm and an exposure time of 0.05 s. A Pilatus 2 M detector, positioned at a sample-to-detector distance of 2 m, was used to obtain the 2D SAXS patterns at each scanning point, while a Pilatus 300kw detector, positioned vertically as a strip beneath the sample, was employed for WAXS, with a distance of 0.63 m from the sample holder. This detector configuration covers a q range from approximately 0.03 to 5 nm^{-1}, where q is the scattering vector defined as $q = 4\pi\ \lambda\ \sin(\theta)$, with λ denoting the X-ray wavelength and θ representing the half scattering angle. Inside the flight tube, a 1.5 mm steel beam stop was installed to block the direct beam and protect the detector from damage. The 2D scattering patterns underwent azimuthal integration and divided the detector into 16 angular segments.

Subsequently, the orientation and degree of orientation were examined within a specific q range (q = 0.007 – 0.083 nm^{-1}), following the methodology outlined by Bunk et al.[4] The symmetric intensity a_0 is thereby defined as the average scattering over all azimuthal angles, the asymmetric intensity a_1 is defined as the amplitude of the second Fourier component, and the degree of orientation was defined as a_1/a_0. A combination Fig. was used to represent the data, where one pixel represents one measured SAXS/WAXS data point. Each pixel is colored according to the main orientation angle of the scattering, which can be read from the inset color wheel. The asymmetric intensity is encoded as saturation and the symmetric intensity by the brightness. Black areas in this representation correspond to low scattering intensity in the selected q-range, and white areas represent high average scattering but no preferred orientation.

WSL Models and Treatment Groups

Polished enamel surfaces were covered with tape to leave a window approximately 1mm wide and 2 mm long on the facial surfaces of each molar. WSLs were then induced on the window using an established protocol[5]. For comparison, we symmetrically divided all WSL windows into two parts; the bottom was protected by an adhesive tape to act as a negative control, and the top was used for applying the repair treatment and was pre-etched with 15% HCl for two minutes to open up the enamel pores and ensure the infiltration of treatment and were washed with

deionized water, sonicated for 2 minutes in water bath to remove any residual contaminants, air dried, and stored at 4 °C until used. WSL blocks were treated with either Ker_5 or resin infiltrant (ICON®, DMG America). Each treatment group was incubated in three storage media: UPW, artificial saliva[6], or mineralization solution[7]. For the keratin treatments, similar fabrication procedures were undertaken as mentioned previously and were pipetted (20 µL) on the uncovered area to infiltrate the white spot lesions, then left to dry and subsequently stored at 37°C for 7 days. Enamel-treated blocks were subsequently washed with UPW, sonicated for 2 minutes to remove debris, and air-dried.

Bright Light Microscopy Grayscale Pixel Analysis

Teeth samples were analyzed using white light microscopy (VHX-7000 series, Keyence, America) to analyze the lesion surface. Images were obtained using an E20 lens at x20 magnification. Images were then subsequently converted to 8-bit grayscale using Photoshop CS6 (Adobe Photoshop®) and transferred to ImageJ® (NIH). A 400x300 pixel oval selection area was outlined to be used for grayscale data capture as this selection area captured enough pixel information without being influenced by the borders or transition surfaces of the lesion. The selection area was dragged over each area of interest, and grayscale values were recorded for each pixel.

Optical Coherence Tomography (OCT) Analysis

OCT (VivoSight®, Kent, UK) was used to analyze the enamel lesion depth and density. The scan area was 6mm wide to include the entire lesion cross-section with a 0.0001 mm interval between each slice. Two-Dimensional cross-sectional images of the control and intervention areas were captured for each sample. Images were then subsequently transferred to ImageJ (NIH, US) for grayscale analysis. Both pre- and post-intervention images were formed into an image stack and a 100µm scale calibrated within this. The WSL control area was traced using the polygon tool and used as a fixed area to obtain grayscale values for the control and intervention areas of the lesion.

Microhardness Measurements.

Surface microhardness was carried out on all samples by a microhardness tester fitted with a Knoop diamond indenter using the microhardness tester (Duramin-20,

Struers Ltd, Rotherham, UK). Microhardness measurements of sound enamel were obtained from the polished enamel around the WSLs window within the experimental groups. For each sample, five indentations were measured with a spacing of at least 100 μm at load of 0.2 N. After the indentation had been prompted, samples were imaged on white light microscope (VHX-7000 series, Keyence, America), and microhardness measurements were then calculated from the indents using the formula below.

HK = 14.299 x F / D2, *F* = the applied force (N), and *D* = the large diagonal (mm)

Nanoindentation Measurements

Keratin films and enamel samples were glued onto an aluminum holder. Nanoindentation tests were carried out by nanoindenter (iNano) by Nanomechanics, Inc. (maximum indentation load of 50 mN), which was used in dynamic mode in order to monitor the variation of the Young's modulus and Hardness as a function of the indentation depth. The mechanical properties of the mineralized films were recorded with an indentation depth of 30 nm on the films in order to decrease the organic tissue influence underneath. Enamel samples Young's modulus and Hardness were evaluated following a previously established method[8] with a maximum depth of 1000 nm with 50-100 indents made for each sample.

SUPPLEMENTARY FIGURES

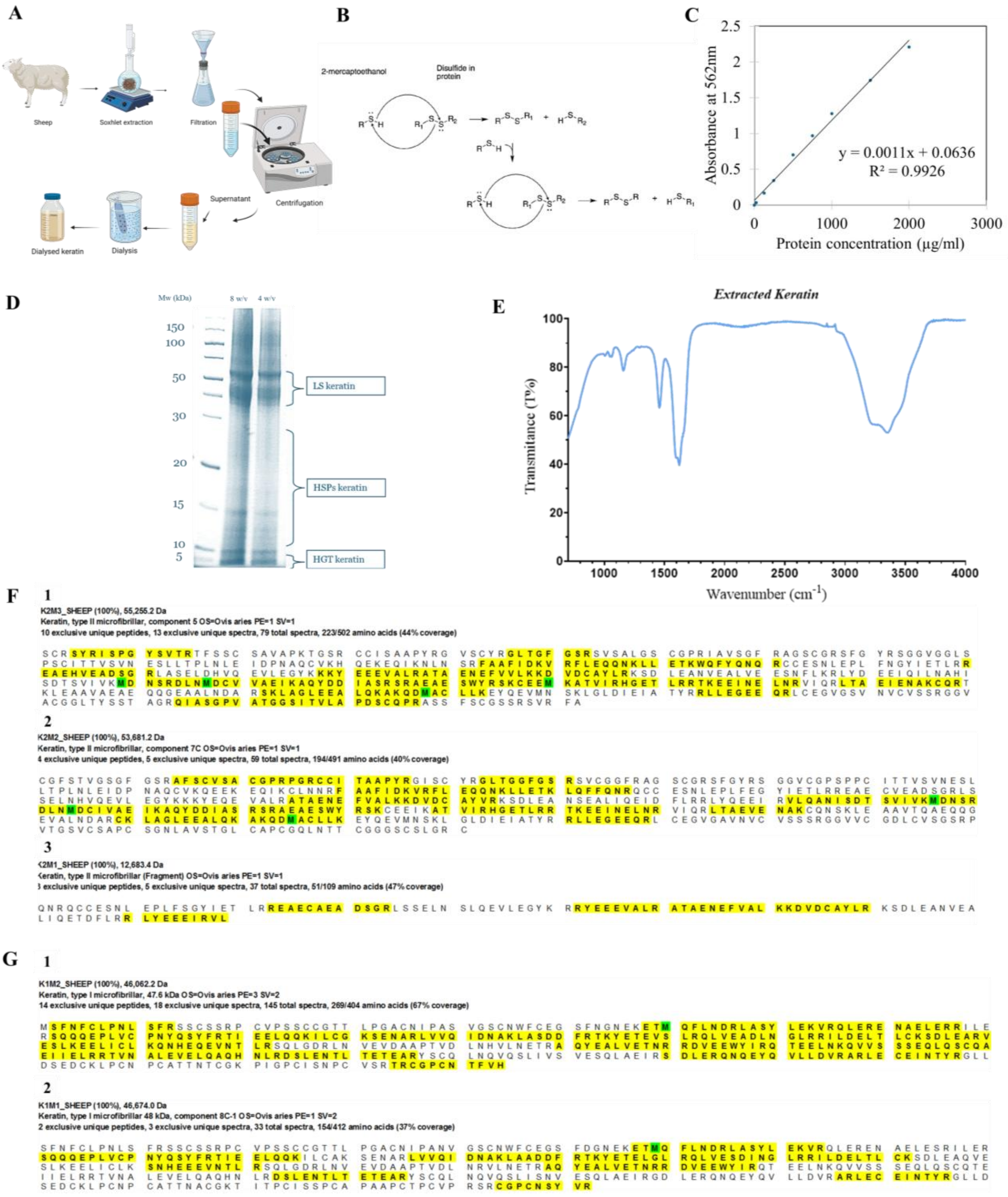

Supplementary Figure 1: Keratin extraction characterization.

A Keratin extraction process illustration. **B** Mechanistic overview of the disulfide bond cleavage, in wool fibers. Reduction reaction occurs between sulfur bridged cystine residues and 2-mercaptoethanol. **C** SDS-PAGE of keratin aqueous solutions at concentrations 8 & 4 w/v, respectively, demonstrating different keratin type bands. **D** BCA showing the absorbance readings vs. concentration to find a linear fit regression line, the equation is used to calculate

the concentration of keratin. **E** ATR-FTIR of keratin during the different extraction and purification steps. **F** LC-MS/MS analysis showing sequence coverage of the proteins clustered as the most abundant identification in Band A. Keratin type II microfibrillar proteins were clustered as the most abundant. **G** Database assigned protein identifications for Band B. Keratin type I microfibrillar proteins were clustered as the most abundant. Correct peptide assignments are highlighted in yellow; modified residues are highlighted in green.

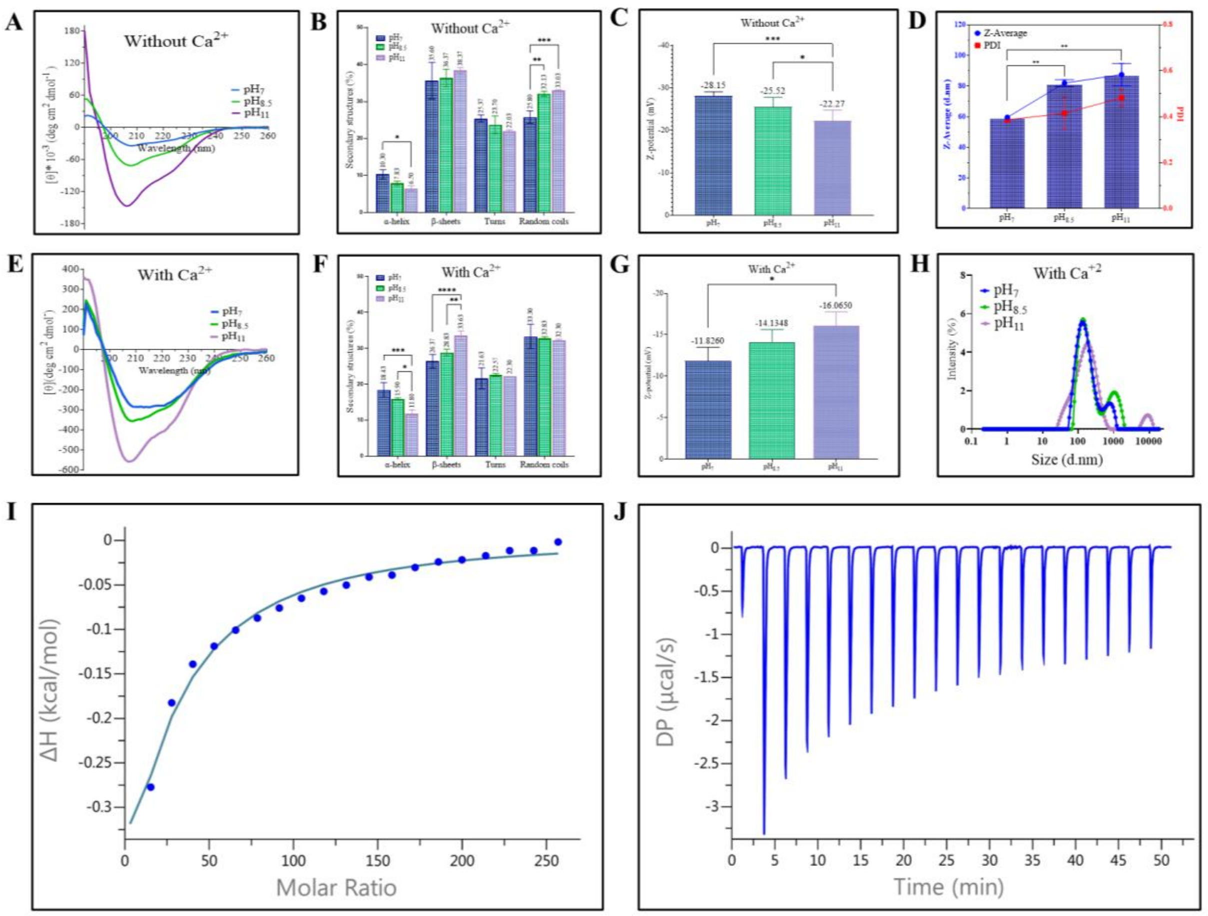

Supplementary Figure 2. Characterization of Keratin in solution with and without adding Calcium.

Characterization of Keratin aqueous solutions at pH 7, 8.5, and 11 without adding calcium ions; **A** CD spectra dataset, **B** Percentage of the Keratin 2ry structures after deconvolution of the CD data using Dichroweb® software, **C** Z-potential, **D** Z-Average and polydispersity index of the particles. Characterization of Keratin aqueous solutions at pH 7, 8.5, and 11 after adding calcium ions, **E** CD spectra, **F** Percentage of the Keratin 2ry structures after deconvolution of the CD data using Dichroweb® software, **G** Z-potential, **H** Particle size distribution analysis. **ITC analysis of calcium binding to keratin at pH 7 demonstrating; I** Raw heat flow showing progressively decreasing exothermic peaks upon injection. **J** Integrated enthalpy change yielded a dissociation constant (Kd) of 2.85 mM, stoichiometry (N) of 1.0, and enthalpy change (ΔH) of –25.0 kcal/mol, indicating moderate-affinity, enthalpy-driven binding of calcium ions to keratin. Significance (*) at $p < 0.05$, n=3

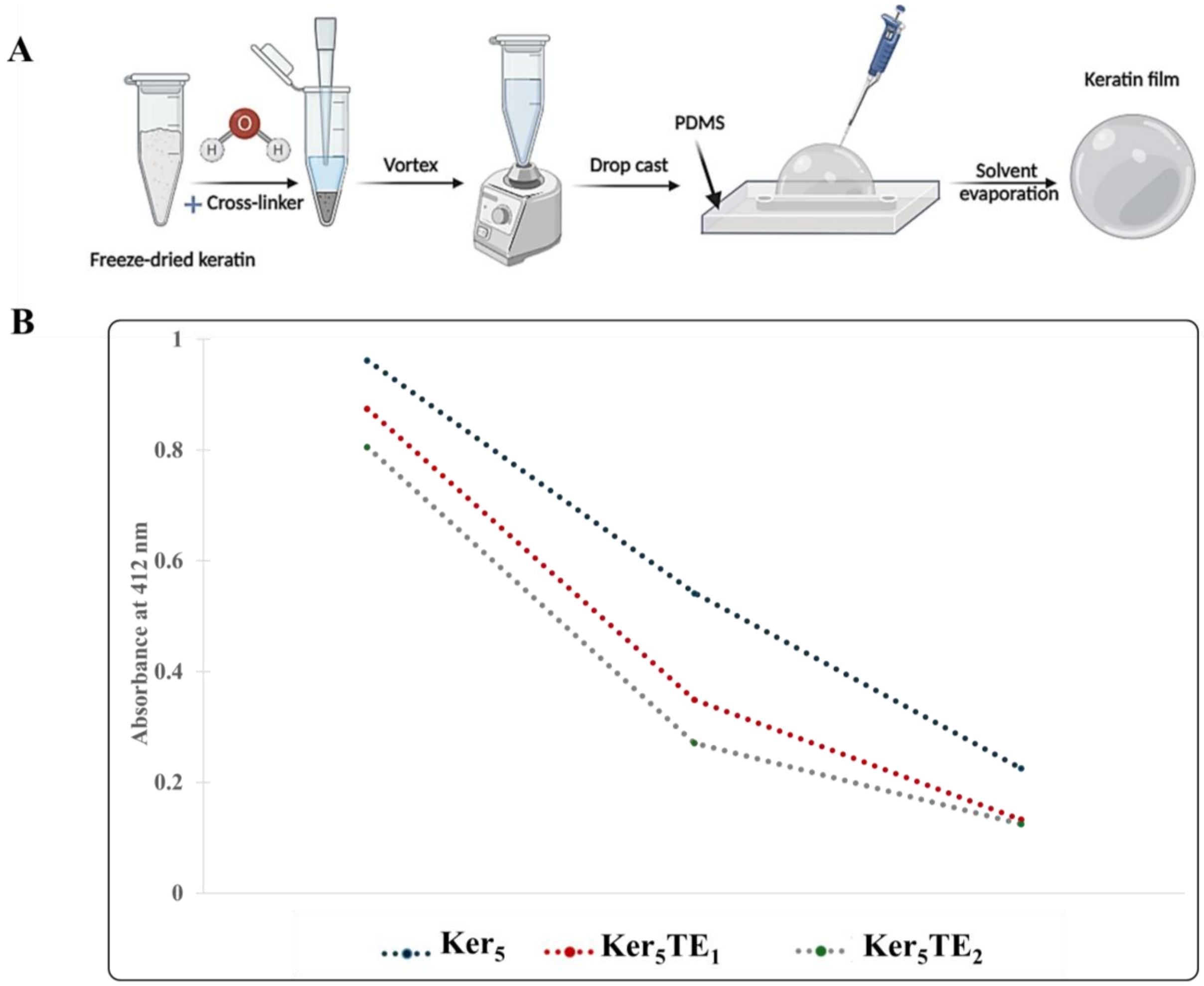

Supplementary Figure 3: Keratin films fabrication and self-assembly.

A illustration showing keratin films' fabrication. **B** Quantitative Ellman's assay absorbance at 412 nm showing keratin samples absorbance at baseline, after 30 minutes and 1 hour, demonstrating amount of the free thiols in the different time points of the films' formation.

Supplementary Table 1: Overview of the fabricated keratin films at pH 7 and their corresponding qualitative assessment criteria and scores. The scoring system is defined as follows: Score 1 – High, Score 2 – Moderate, Score 3 – Mild, and Score 4 – Low.

Keratin Films Qualitative Assessment **(pH7)**		**Ker$_{3}$**	**Ker$_{5}$**	**Ker$_{10}$**
	TE$_{0}$	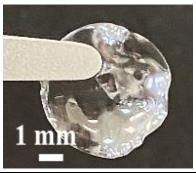	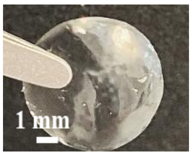	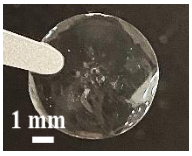
	Ease of Handling	2	3	4
	Collapse potential	3	2	4
	Brittleness	2	3	4
	Transparency	1	1	1
	TE$_{0.5}$	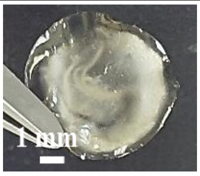	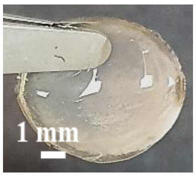	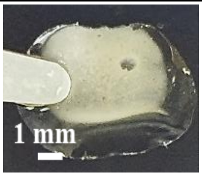
	Ease of Handling	2	2	3
	Collapse potential	2	3	3
	Brittleness	1	2	3
	Transparency	2	2	2
	TE$_{1}$	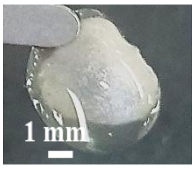	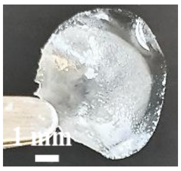	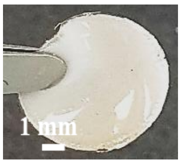
	Ease of Handling	3	4	3
	Collapse potential	3	3	4
	Brittleness	3	3	3
	Transparency	3	3	4
	TE$_{2}$	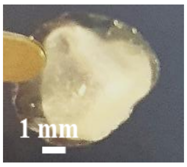	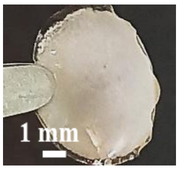	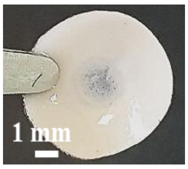
	Ease of Handling	3	4	3
	Collapse potential	3	3	4
	Brittleness	3	3	3
	Transparency	3	4	4

Supplementary Table 2: Overview of the fabricated keratin films at pH 11 and their corresponding qualitative assessment criteria and scores. The scoring system is defined as follows: Score 1 – High, Score 2 – Moderate, Score 3 – Mild, and Score 4 – Low..

Keratin Films Qualitative Assessment **(pH11)**		**Ker$_3$**	**Ker$_5$**	**Ker$_{10}$**
	TE$_0$	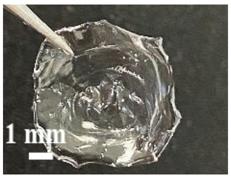	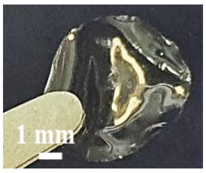	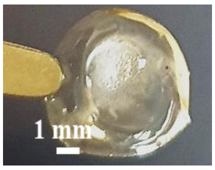
	Ease of Handling	1	2	3
	Collapse potential	1	2	3
	Brittleness	1	2	3
	Transparency	1	1	1
	TE$_{0.5}$	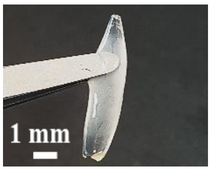	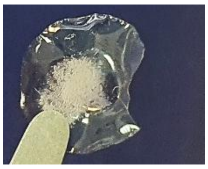	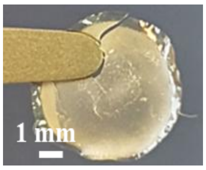
	Ease of Handling	1	1	2
	Collapse potential	1	2	3
	Brittleness	1	2	3
	Transparency	1	2	3
	TE$_1$	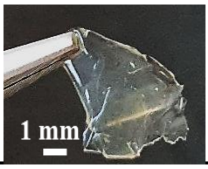	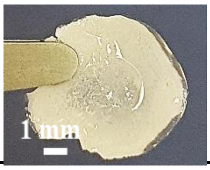	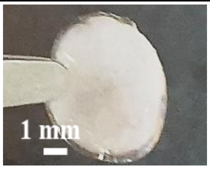
	Ease of Handling	2	3	3
	Collapse potential	1	2	2
	Brittleness	2	2	3
	Transparency	2	3	4
	TE$_2$	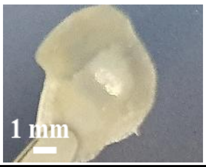	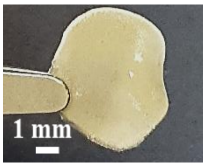	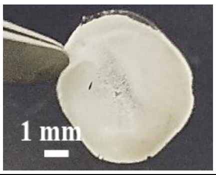
	Ease of Handling	3	3	4
	Collapse potential	2	3	4
	Brittleness	2	2	2
	Transparency	3	4	4

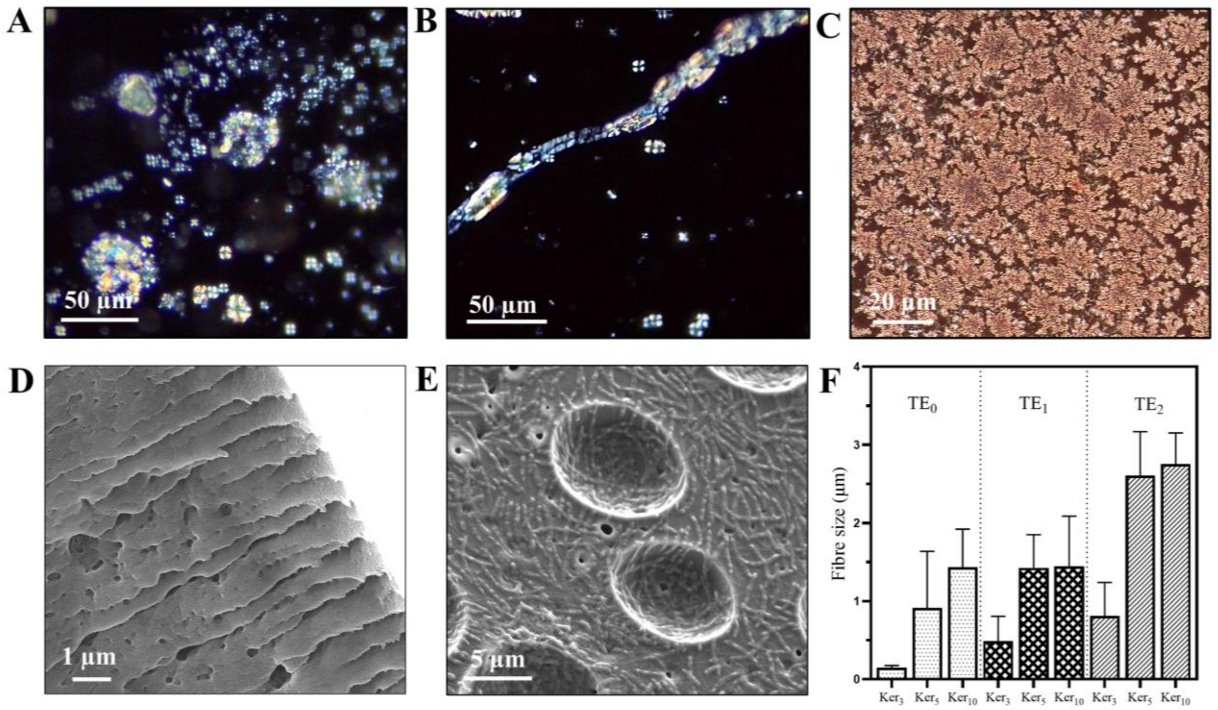

Supplementary Figure 4: Keratin films fabrication & characterization.

A, B Polarized light microscope showing spherulite organization patterns. **C** Congo red staining of a keratin film demonstrating organic dendritic structures. SEM of keratin films (3 w/v %) showing their assembly into nanofibrils **D** Self-crosslinked (Ker_3), and **E** Keratin crosslinked with TEGDMA (Ker_3TE_1). **F** Fibril quantification of Keratin films (TE_0 represents self-crosslinked keratin, TE_1: Keratin crosslinked with TEGDMA (0.4 w/v%), and TE_2: Keratin crosslinked with TEGDMA (0.8 w/v%).

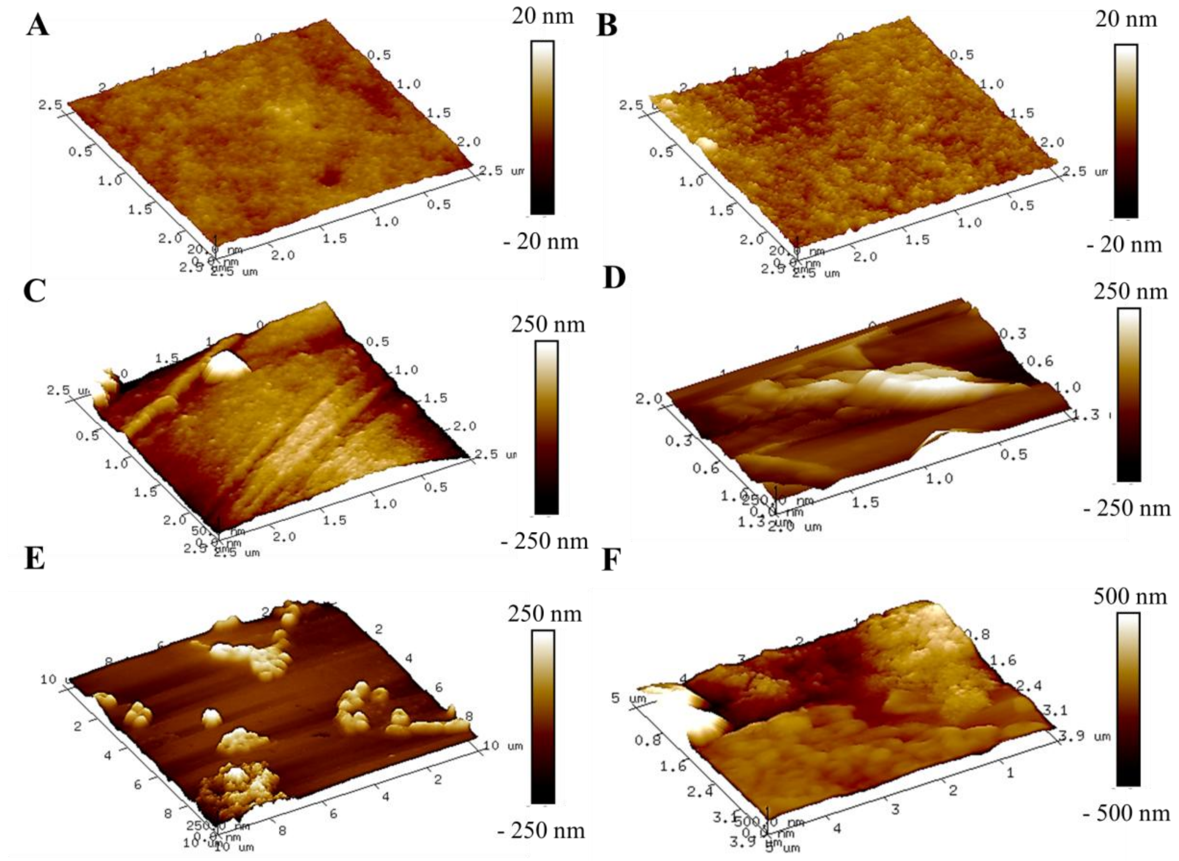

Supplementary Figure 5: Keratin characterization by AFM before and after mineralization.

AFM of un-mineralized keratin at: A 5 w/v %, demonstrating their assembly in solution into nanospheres of average diameter 24.2 ± 5.3nm, and **B** 10 w/v %, revealing an increase in the diameter of these nanospheres to 33.0 ± 6.9 nm, these nanospheres are believed to serve as scaffolds for mineral nucleation. **AFM of keratin-mediated mineralization over a 7-day period reveal the following morphological changes; C** Nanospheres densely align in rows, **D** These nanospheres further assemble into larger structures. **E** Subsequently, the assemblies break down into smaller spheres, which then **F** thickens and fuse to form mineralized apatite.

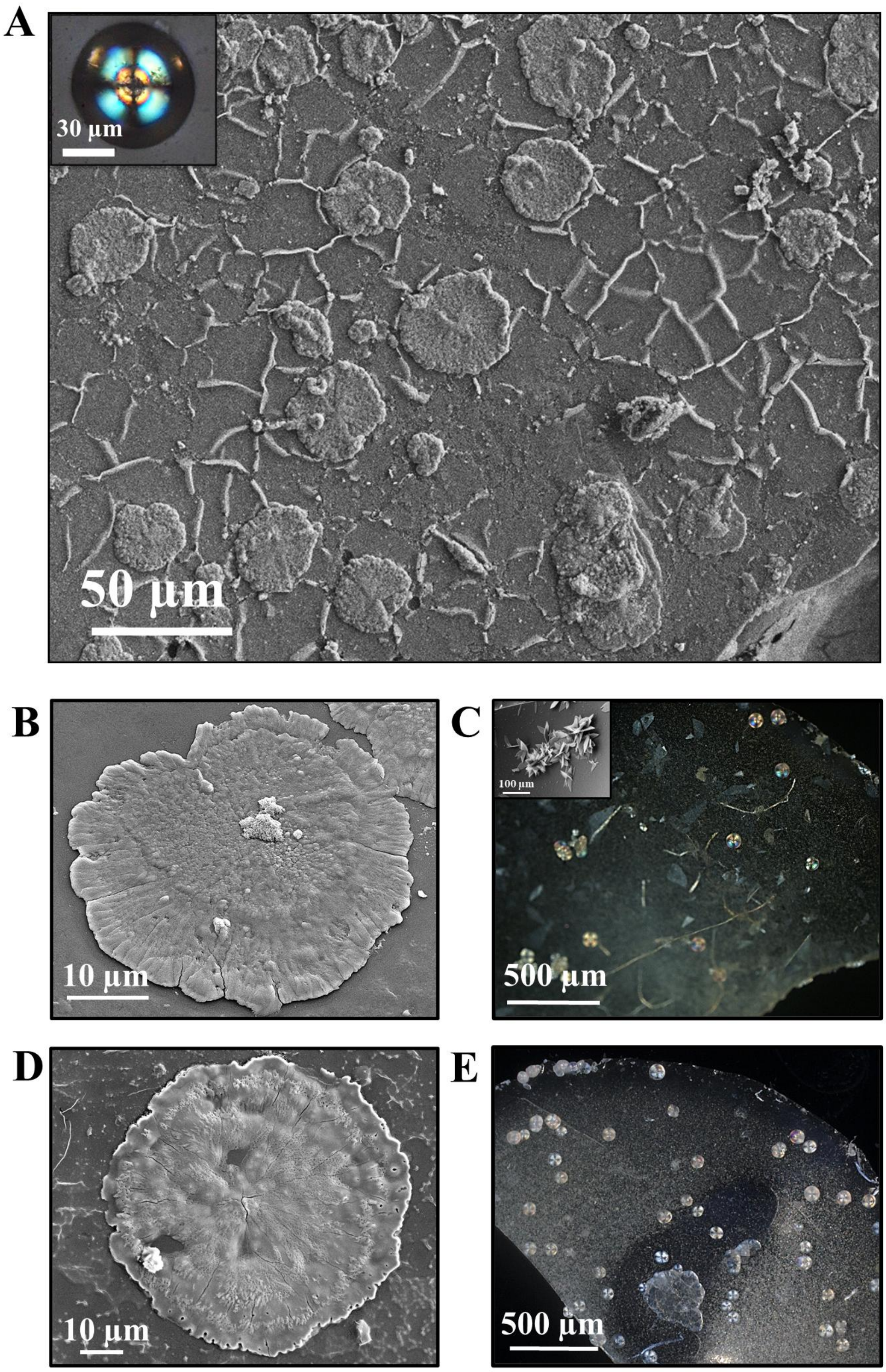

Supplementary Figure 6: Keratin mineralization pattern over time.

A SEM of a mineralized keratin film showing mineralized spherulites growing on the surface, *inset display an inorganic spherulite under polarized filters demonstrating their size increase after mineralization. **B** Keratin mineralized spherulite on day 1 of mineralization. **C** Light microscope image of spherulites under polarized light at day 1, * Inset showing SEM of platelet-like crystals. **D** Keratin mineralized spherulite on day 2 of mineralization. **E** Polarized light microscope image of spherulites on day 2.

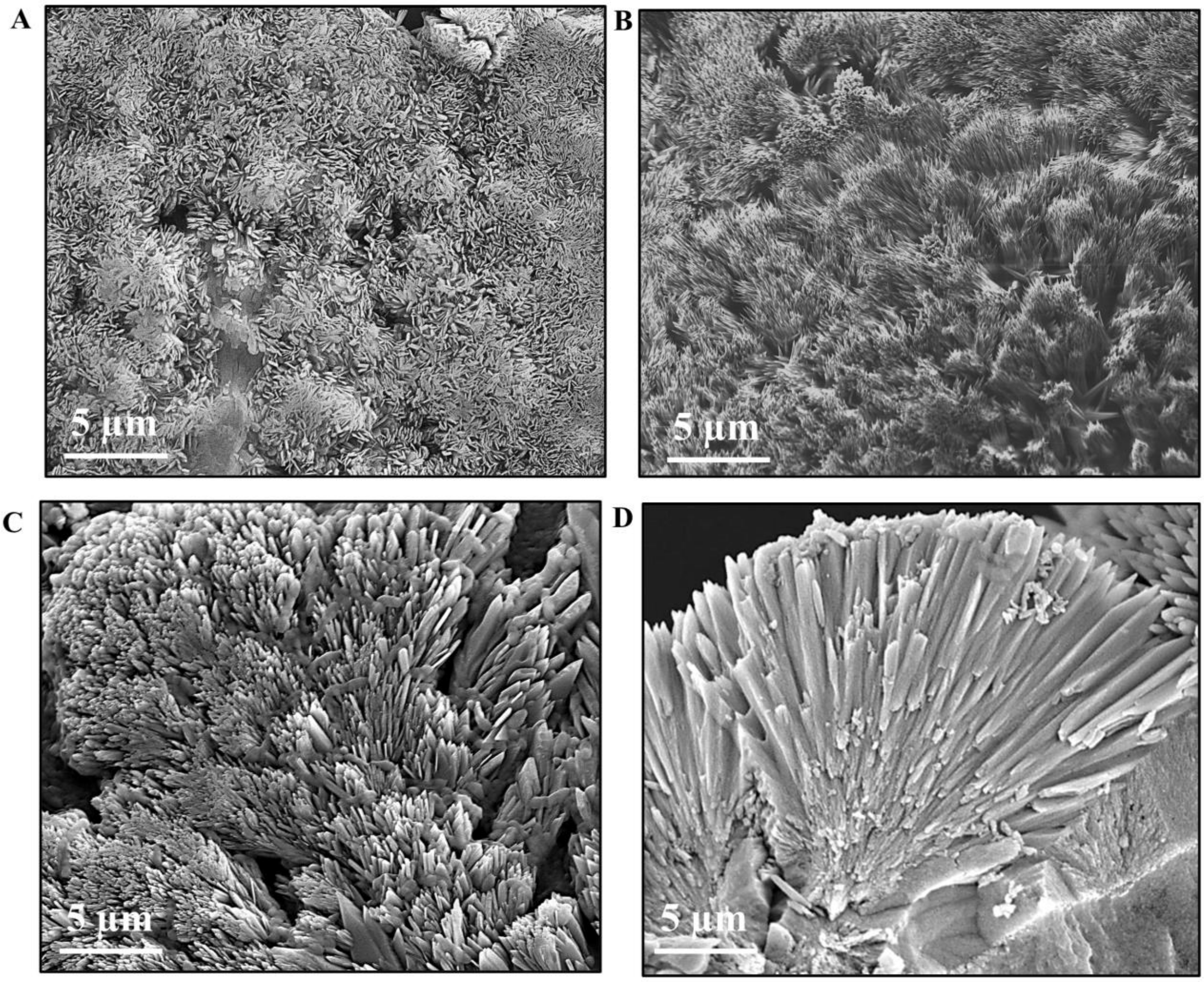

Supplementary Figure 7. Time Time-dependent mineralization patterns on keratin films. SEM images showing morphological evolution over time. **A** By day 7 well-defined, mineral deposition spreads across the film surface, **B** Structures become denser and more needle-like. **C** On day 14, larger merged spherulites with fine nanocrystalline matrices appear. **D** By day 30, extensive networks of radially oriented, needle-like crystals had developed, exhibiting progressive increases in size and surface coverage.

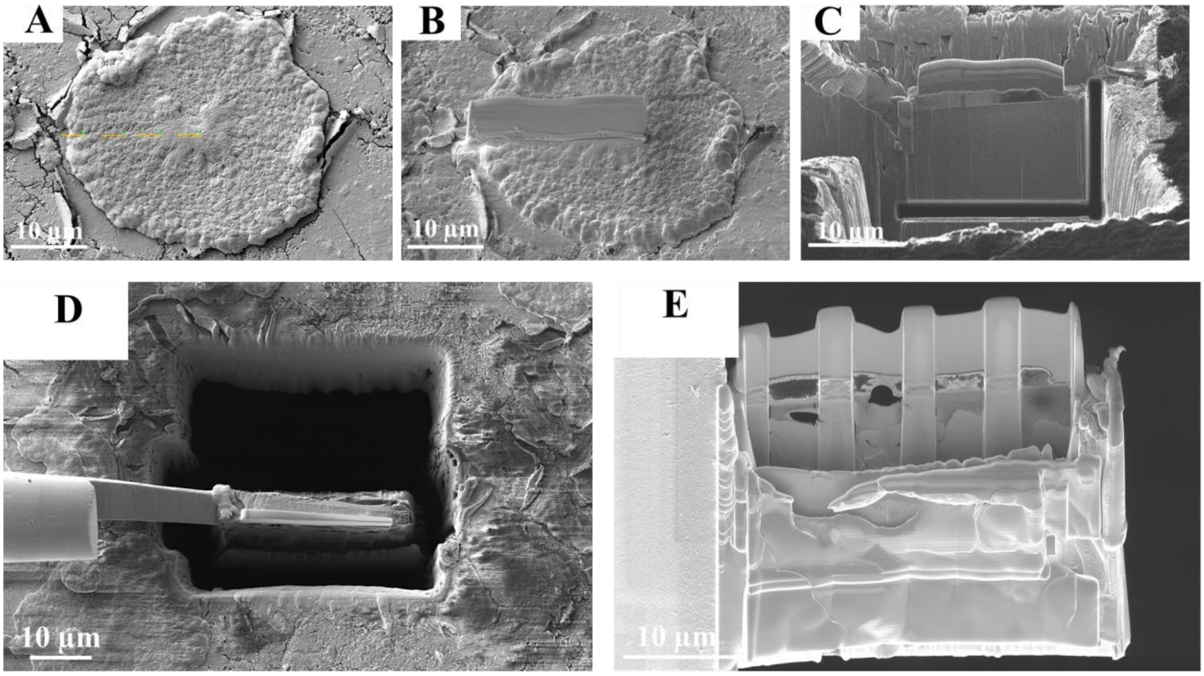

Supplementary Figure 8: FIB lift out and milling.

A Four windows (~3um wide each) chosen along a line at the target location. **B** Tungsten deposited with ion beam deposition to protect the sample throughout the FIB process. **C** Trenches milled around the lamella. **D** Lamella extracted with a micromanipulator. **E** Four windows cleaned by low voltage to minimize amorphization and gallium implantation.

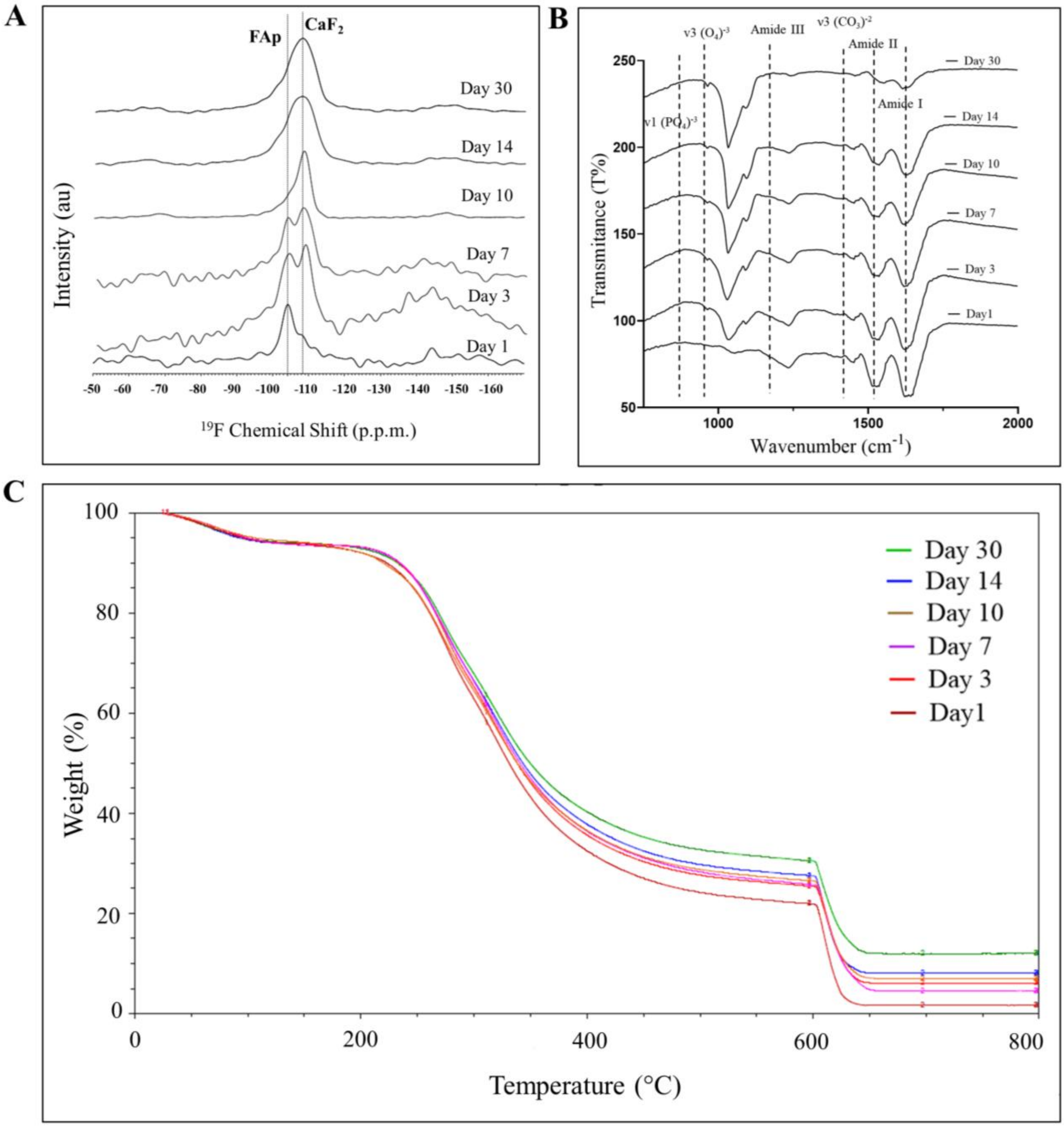

Supplementary Figure 9: Keratin film mineralization over 30 days.

A ^{19}F solid-sate MAS-NMR of mineralized keratin films collected over 30 days, confirming the presence of FAp and CaF_2 phase at – 103 and – 108 p.p.m., respectively. A predominant FAp peak is observed at day 1, while CaF_2 emerges by days 3 and 7, becoming progressively more prominent by Day 10 and dominates the spectra by Days 14 and 30. **B** ATR-FTIR spectra confirm progressive mineralization over time, with increasing intensity of phosphate vibration bands indicative of apatite formation. Concurrently, the gradual decrease in the intensity of amide I, II, and III bands reflects the reduction of organic content as mineral deposition within the keratin matrix increase. **C** TGA curve of mineralized keratin film (Sample 1-KCL) showing

~10% weight loss below 150°C (water loss), ~55% loss between 200–500°C (keratin decomposition), and a plateau above 650°C indicating stable inorganic mineral phases. Residual mass increased with mineralization time, reflecting progressive mineral deposition from Day 1 to Day 30.

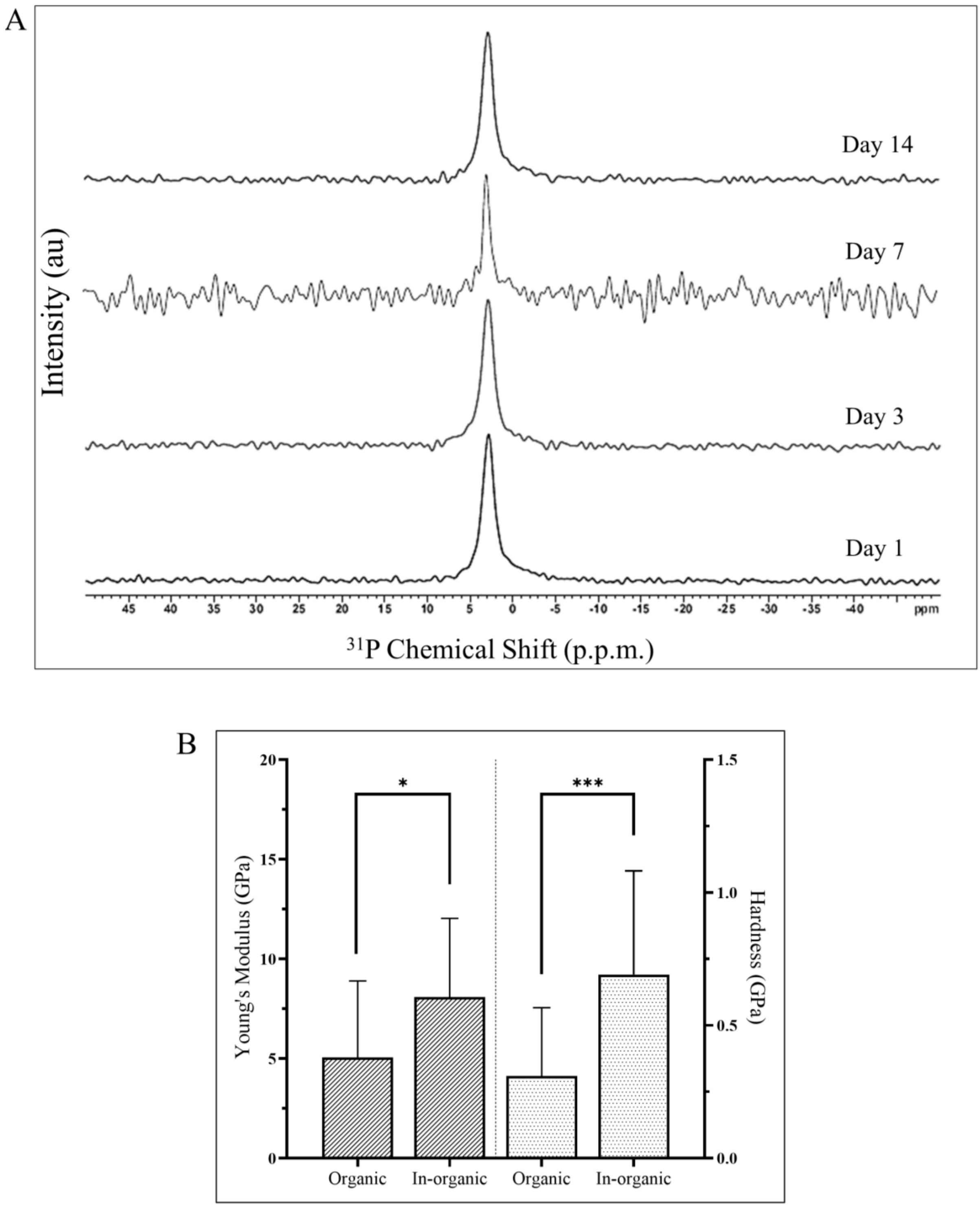

Supplementary Figure 10: Physico-mechanical characterization of keratin film mineralization.

A ^{31}P MAS-NMR chemical shift demonstrates the presence of apatite peak at ~ 3 ppm in the keratin films. **B** Young's modulus and Hardness measurements of Keratin films before and after mineralization demonstrates increase of both features after mineralization, where significance (*) at p <0.05, n=3.

Supplementary Table 3: Table displaying the influence of different factors e.g. protein concentration and crosslinking on the aqueous keratin secondary structures' conformation before drying.

2ry structures Group	IMβ	β-sheets	Random coils	α-helix	β-turn	Random: β
**Ker_3**	14.52	29.47	8.99	10.02	37	0.2
	10.46	33.76	12.65	11.36	31.78	0.29
	21.17	25.11	14.21	15.34	24.181	0.31
**Average SD**	**15.38 5.41**	**29.45 4.33**	**11.95 2.68**	**12.24 2.77**	**30.987 6.45**	**0.27 0.05**
**Ker_5**	24.32	15.79	24.45	15.93	19.51	0.61
	21.78	11.61	29.46	8.32	28.83	0.88
	19.66	18.6	21.08	16.33	24.33	0.55
**Average SD**	**21.92 2.33**	**15.33 3.52**	**25 4.22**	**13.53 4.51**	**24.22 4.66**	**0.68 0.17**
**Ker_{10}**	21.7	8.83	24.14	16.58	28.75	0.79
	19.03	11.07	24.2	18.63	27.07	0.8
	18.63	9.23	25.13	20.01	27	0.9
**Average SD**	**19.79 1.67**	**9.71 1.195**	**24.49 0.56**	**18.41 1.73**	**27.61 0.99**	**0.83 0.06**
**Ker_3TE_1**	28.43	24.1	18.8	8.04	20.63	0.36
	15.35	24.78	15.82	9.99	34.06	0.4
	22.98	26.49	10.2	12.52	27.83	0.21
**Average SD**	**22.25 6.57**	**25.12 1.23**	**14.94 4.37**	**10.18 2.25**	**27.51 6.72**	**0.32 0.1**
**Ker_5TE_1**	20.59	9.08	27.9	11.77	30.66	0.94
	13.15	21.87	22.4	17.55	25.03	0.64
	22.46	16.27	19.86	20.01	21.4	0.51
**Average SD**	**18.73 4.92**	**15.74 6.41**	**23.39 4.11**	**16.44 4.23**	**25.7 4.67**	**0.69 0.22**
**$Ker_{10}TE_1$**	14.07	8.7	30.1	19.62	27.51	1.32
	15.87	10.91	28.98	22.56	21.68	1.08
	13.9	8.71	33.72	17.44	26.23	1.49
**Average SD**	**14.61 1.09**	**9.44 1.27**	**30.93 2.48**	**19.87 2.57**	**25.14 3.06**	**1.3 0.21**

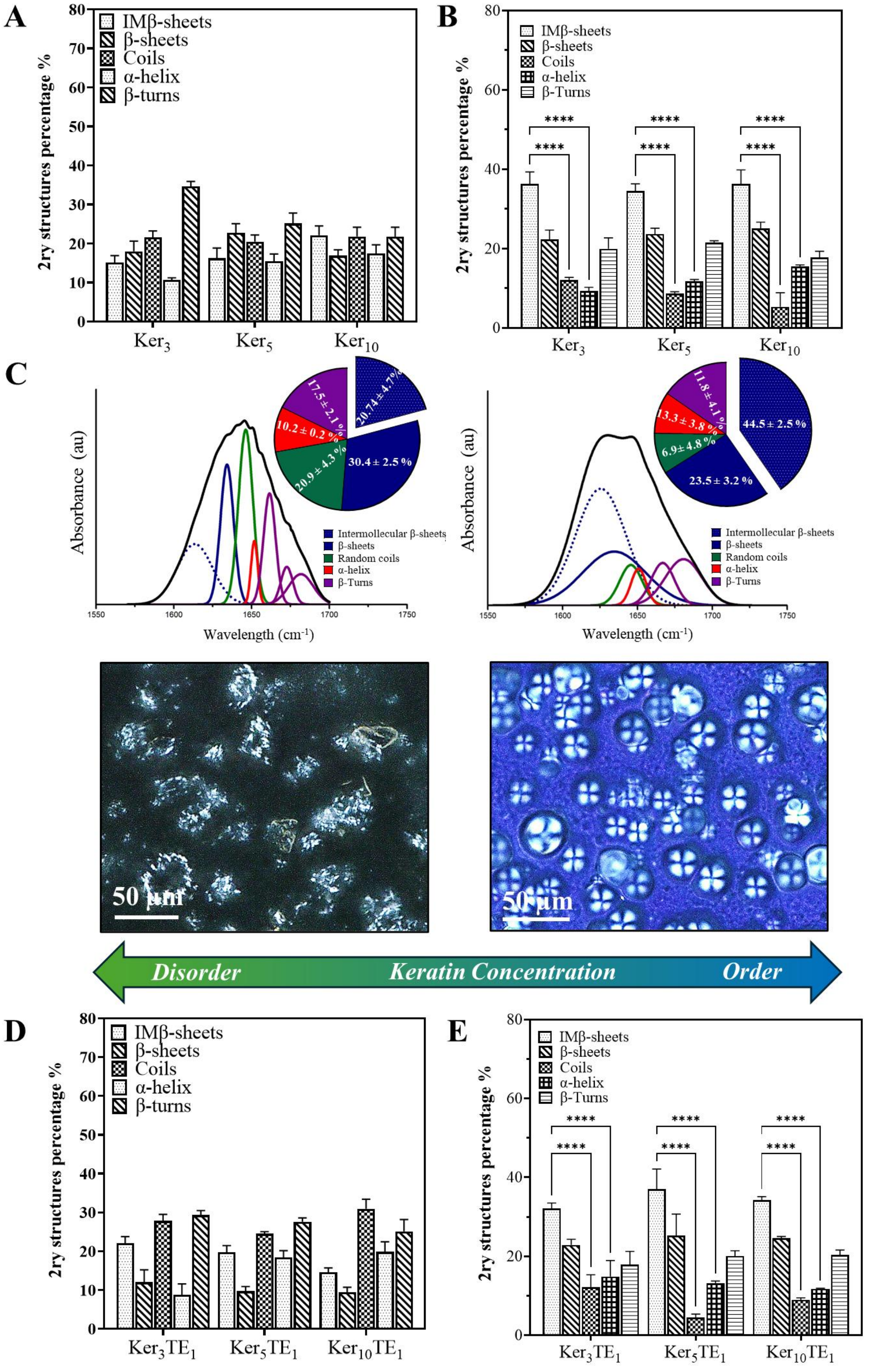

Supplementary Figure 11: Aqueous Keratin secondary structure conformation before and after drying.

A, B Amide I FTIR deconvolution of keratin secondary structures in self-crosslinked keratin before and after drying, respectively, **C** ATR-FTIR deconvolution of the Amide I region for two

different films (Ker_3 and Ker_{10}), demonstrating an increase in β-sheet content and decrease in random coil structures with increasing keratin concentration, alongside corresponding PLM images displaying a more ordered spherulites pattern in higher concentration films. **D, E** Amide I FTIR deconvolution of TEGDMA-crosslinked keratin films (0.4 w/v%) before and after drying, respectively. Statistical differences were considered significant (*) at $p < 0.0001$, $n = 3$

Supplementary Table 4: Table demonstrating the organic-inorganic mechanism and mineralization tuneability indicating the impact of organic matrix conformational change on the mineralization pattern.

Organic matrix conformational impact on mineralization pattern		Ker_3	Ker_5	Ker_{10}
	pH$_7$	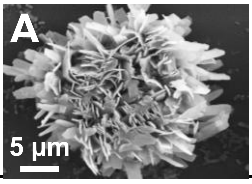	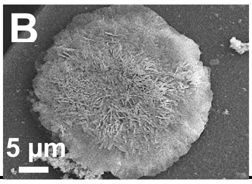	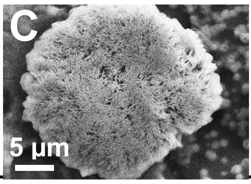
	Random: β-sheets	**0.21 ±0.01**	**0.14 ± 0.01**	**0.1 ± 0.06**
	TE$_{10}$ pH$_7$	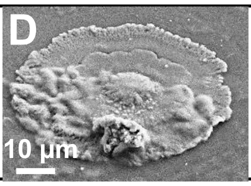	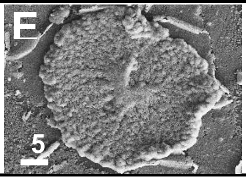	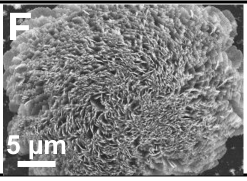
	Random : β-sheets	**0.21 ± 0.05**	**0.1 ± 0.01**	**0.15 ± 0.01**
	TE$_{20}$ pH$_7$	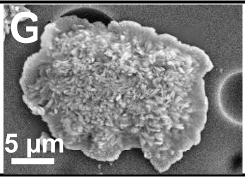	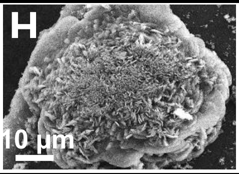	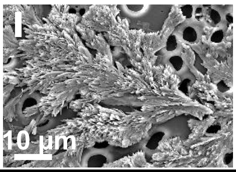
	Random : β-sheets	**0.22 ± 0.02**	**0.18 ± 0.02**	**0.19 ± 0.03**
	pH$_{11}$	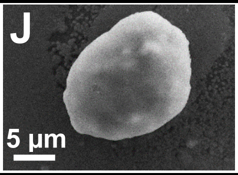	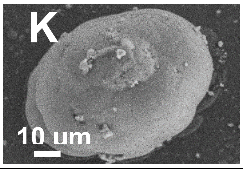	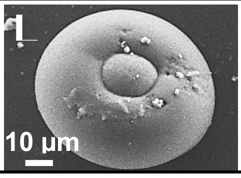
	Random : β-sheets	**0.25 ± 0.03**	**0.24 ± 0.01**	**0.25 ± 0.03**
	TE$_{10}$ pH$_{11}$	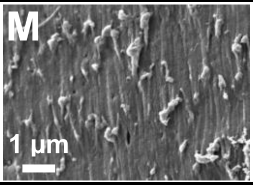	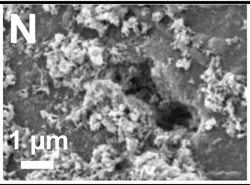	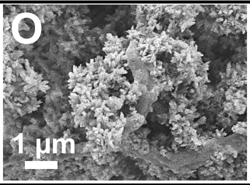
	Random : β-sheets	**0.33 ± 0.06**	**0.39 ± 0.06**	**0.25 ± 0.04**
	TE$_{20}$ pH$_{11}$	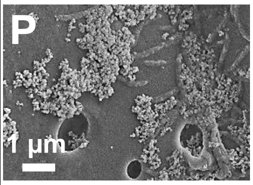	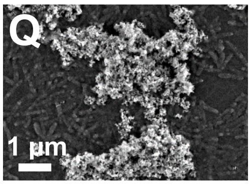	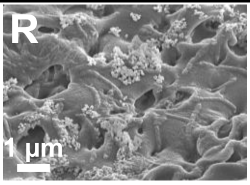
	Random: β-sheets	**0.57 ± 0.2**	**0.52 ± 0.07**	**0.47 ± 0.04**

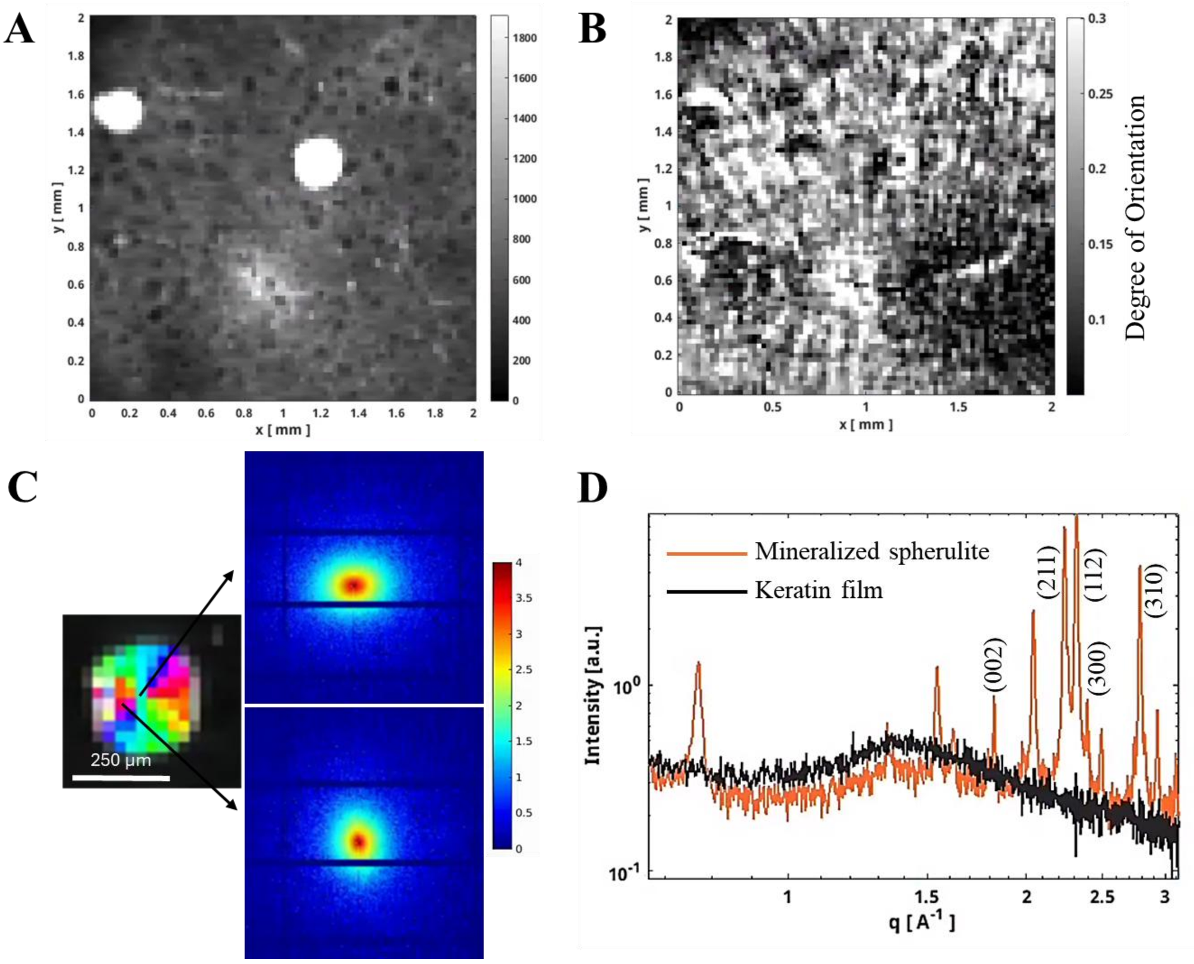

Supplementary Figure 12: SAXS characterization of keratin films.

A SAXS symmetric amplitude figure of the mineralized keratin film in the analyzed q-range of 0.004 – 0.005 Å^{-1}. **B** The degree of orientation in the analyzed q-range of 0.007 – 0.083 Å^{-1}, determined from the asymmetry of the SAXS pattern and calculated for each pixel, is visually represented by the grey scale. Brighter areas indicate a higher degree of orientation. **C** Selected 2D scattering patterns from different positions of the mineralized spherulite. **D** WAXS diagrams of the mineralized spherulite (~ 250 µm) and keratin film.

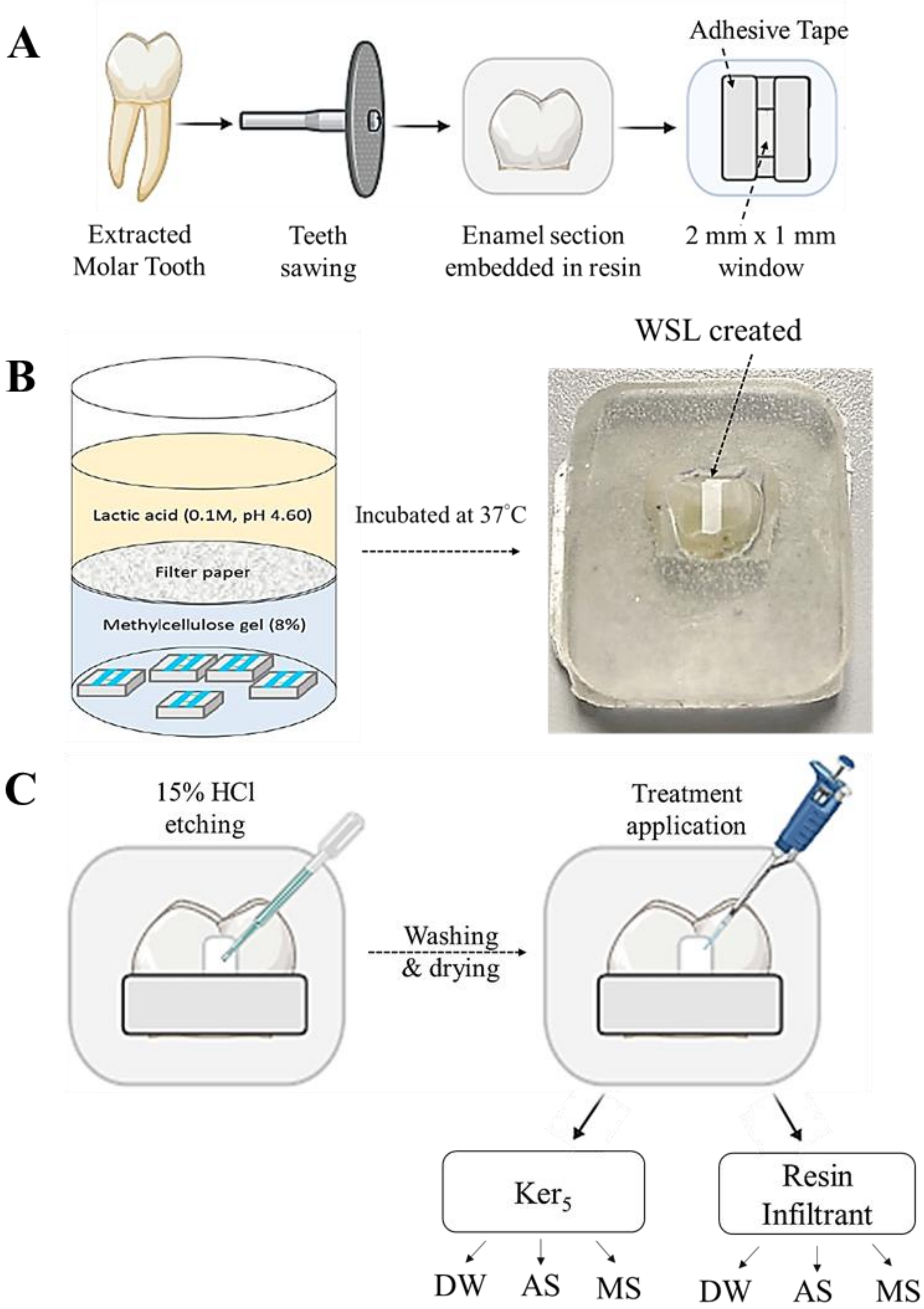

Supplementary Figure 13: Enamel section preparation and WSL induction process illustration. A Enamel section preparation from an extracted molar**. B** Acidic gel preparation for WSL induction and lesion creation after 7 days of incubation in 37ºC. **C** Removing the smear layer with 15% HCl and applying the different treatment groups.

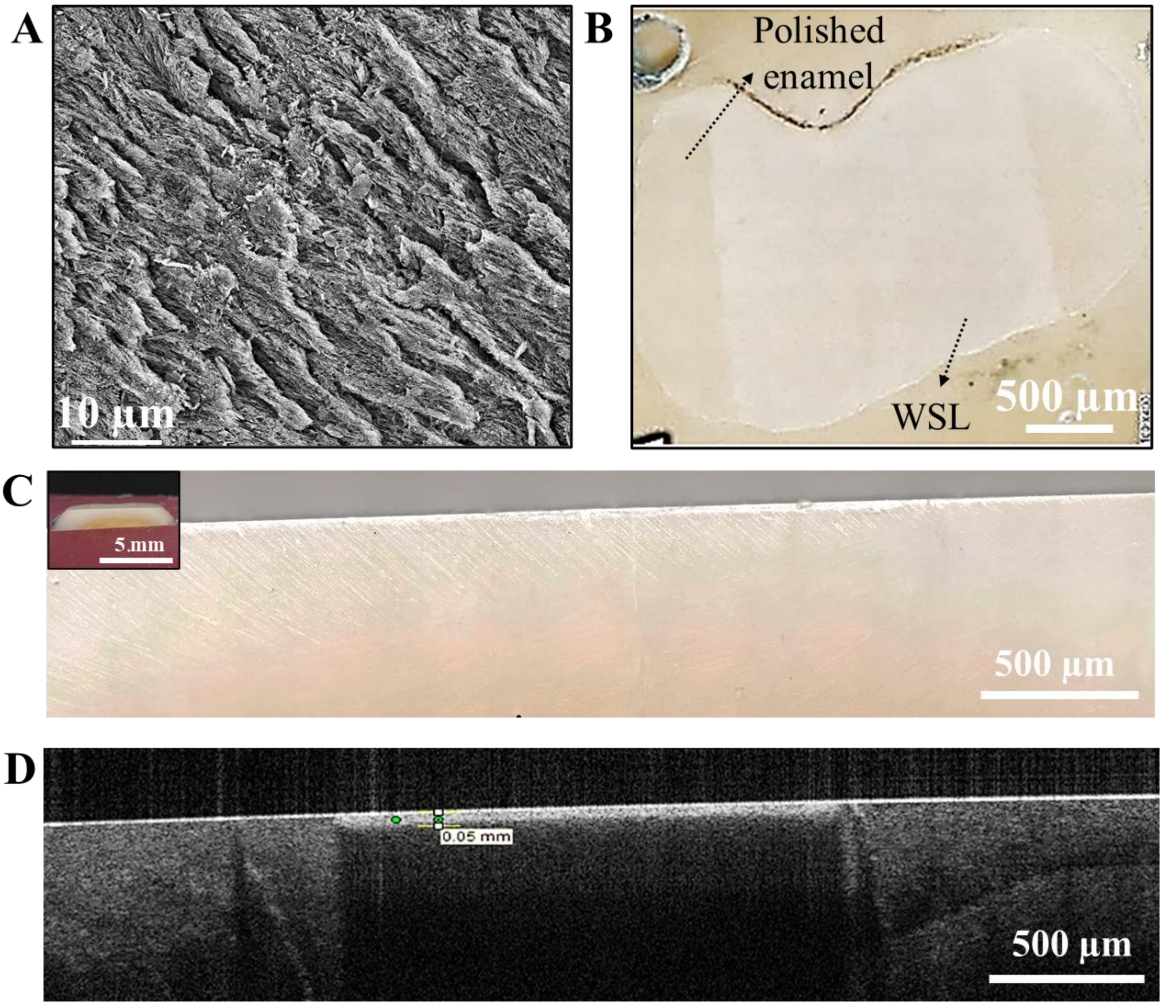

Supplementary Figure 14: WSL characterization. A SEM demonstrating lost enamel prism/ inter-prism structure confirming the induction of WSL at ~50 µm deep, **B** Light microscopy surface view of a demineralized enamel window, **C** Cross-sectional view demonstrating the chalky white appearance on top of native enamel, denoting WSL induction, **D** OCT cross-sectional imaging of artificially induced WSL demonstrating a more intense OCT signal, which is evidence confirming WSL induction.

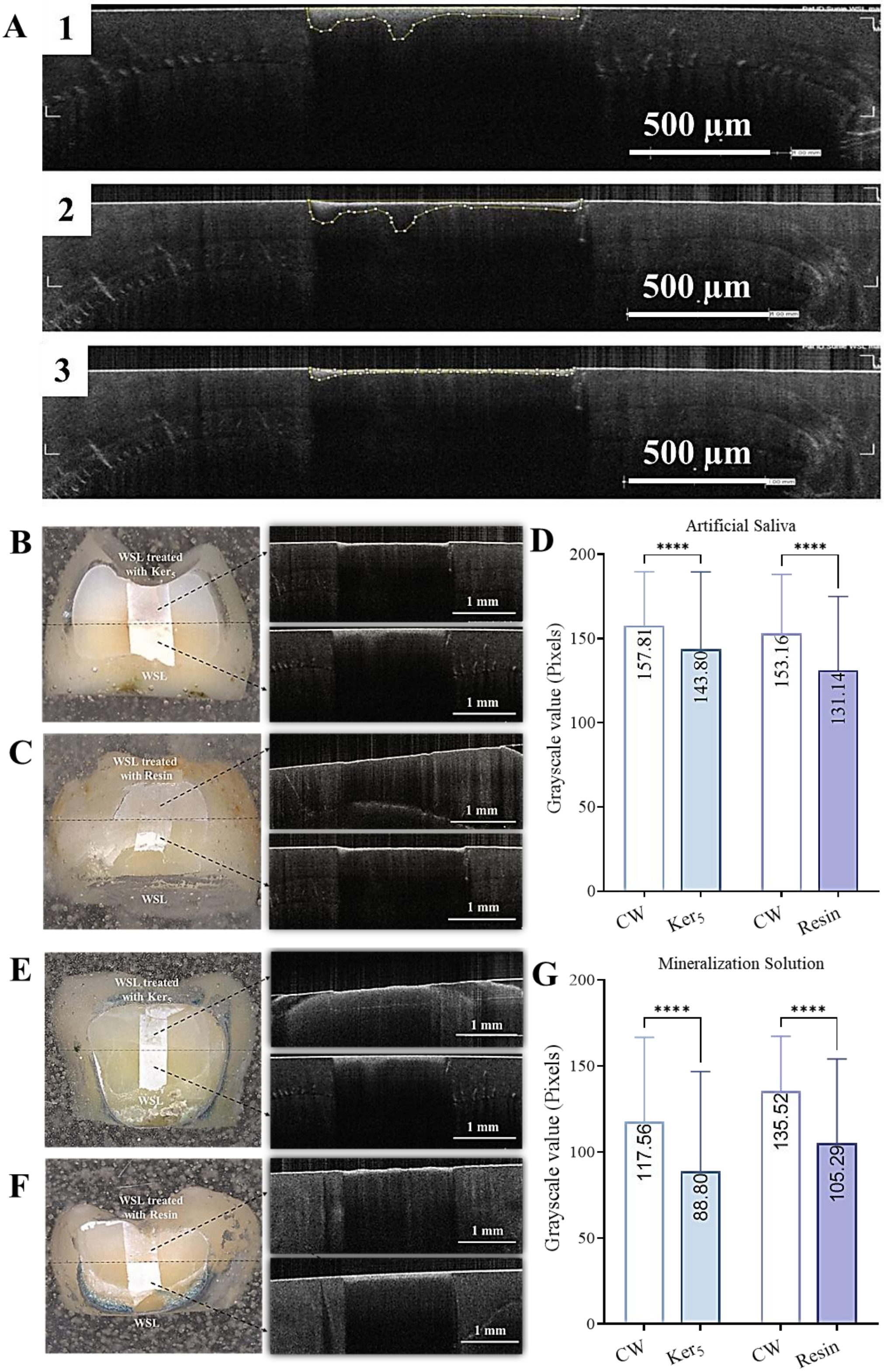

Figure 15: Grayscale pixel analysis of WSL via OCT. (A) WSL grayscale pixel analysis method obtained from OCT images. **1** WSL cross-sectional surface area traced, and grayscale value recorded. **2** Lesion area from A copied across to intervention lesion and grayscale value was recorded. **3** The surface area of the lesion with the intervention was traced and the pixel

difference was recorded. **(B)** Light microscopy image of WSL treated with keratin incubated in artificial saliva and the corresponding OCT scans before and after treatment**. (C)** Light microscopy image of WSL treated with resin incubated in artificial saliva and the corresponding OCT scans before and after treatment**. (D)** Grayscale pixel analysis of both treatment groups in artificial saliva before and after WSL treatment. **(E)** Light microscopy image of WSL treated with keratin incubated in mineralization solution and the corresponding OCT scans before and after treatment**. (F)** Light microscopy image of WSL treated with resin incubated in mineralization solution and the corresponding OCT scans before and after treatment**. (G)** Grayscale pixel analysis of both treatment groups in mineralization solution before and after WSL treatment. **CW:** Control WSL before treatment, **T:** Treated lesion with either Ker_5 or Resin. * Indicates significant difference, where significance at $p < 0.0001$, n=3.

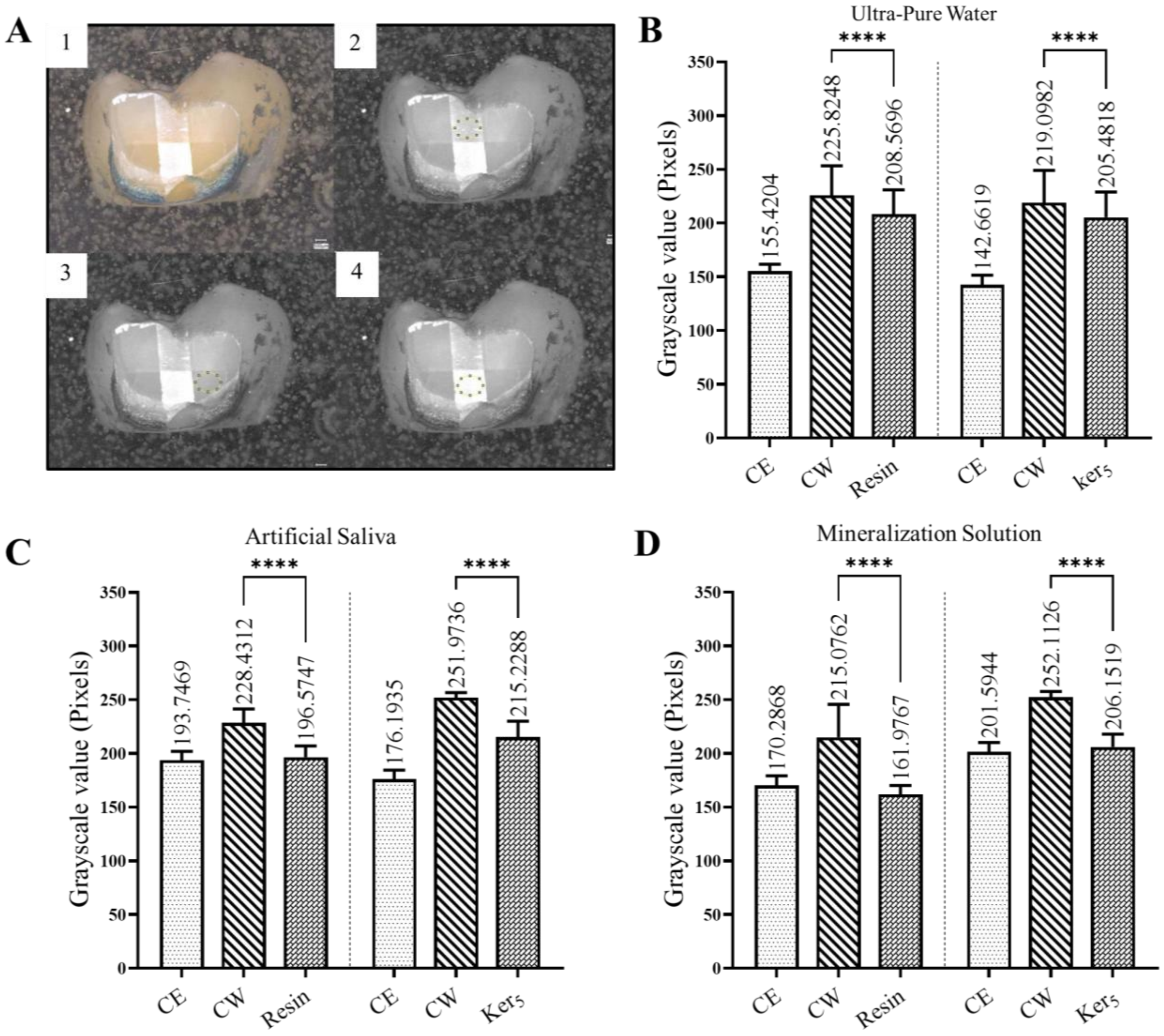

Supplementary Figure 16: WSL characterization with light microscope.

A WSL grayscale pixel analysis method obtained from white light microscope images. 1 Original sample image, 2 Pixel measurements for Enamel window, 3 Pixel measurements for Intervention window, 4 Pixel measurements for WSL window. Graph showing grayscale pixel analysis difference between: CE: enamel (positive control) before WSL induction, CW: enamel after WSL induction, and WSL after treatment intervention with either Ker_5 or Resin in **B** ultra-pure water, **C** artificial saliva, and **D** mineralization solution, demonstrating that keratin treated lesions have shown reduction in WSLs depth and density comparable to the intact enamel before WSL induction and to those treated with resin infiltrant in all media. * Indicates significant difference, where significance at $p < 0.0001$, n=3.

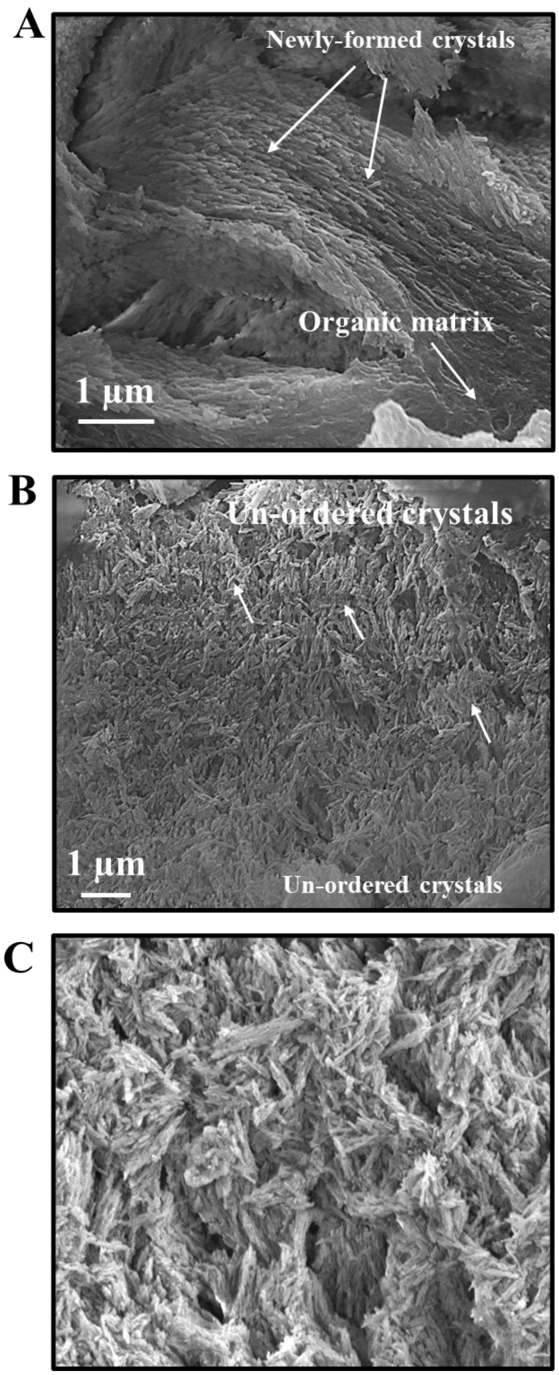

Supplementary Figure 17: Surface characterization of treated enamel lesions.

A Integration between the keratin and the newly-formed enamel-like crystals demonstrating their attachment to the organic matrix which seems to be guiding their growth. Mineralized WSL incubated in **B** artificial saliva and **C** mineralization solution,

arrows pointing on the unorganized pattern that doesn't follow the enamel HAp orientation.

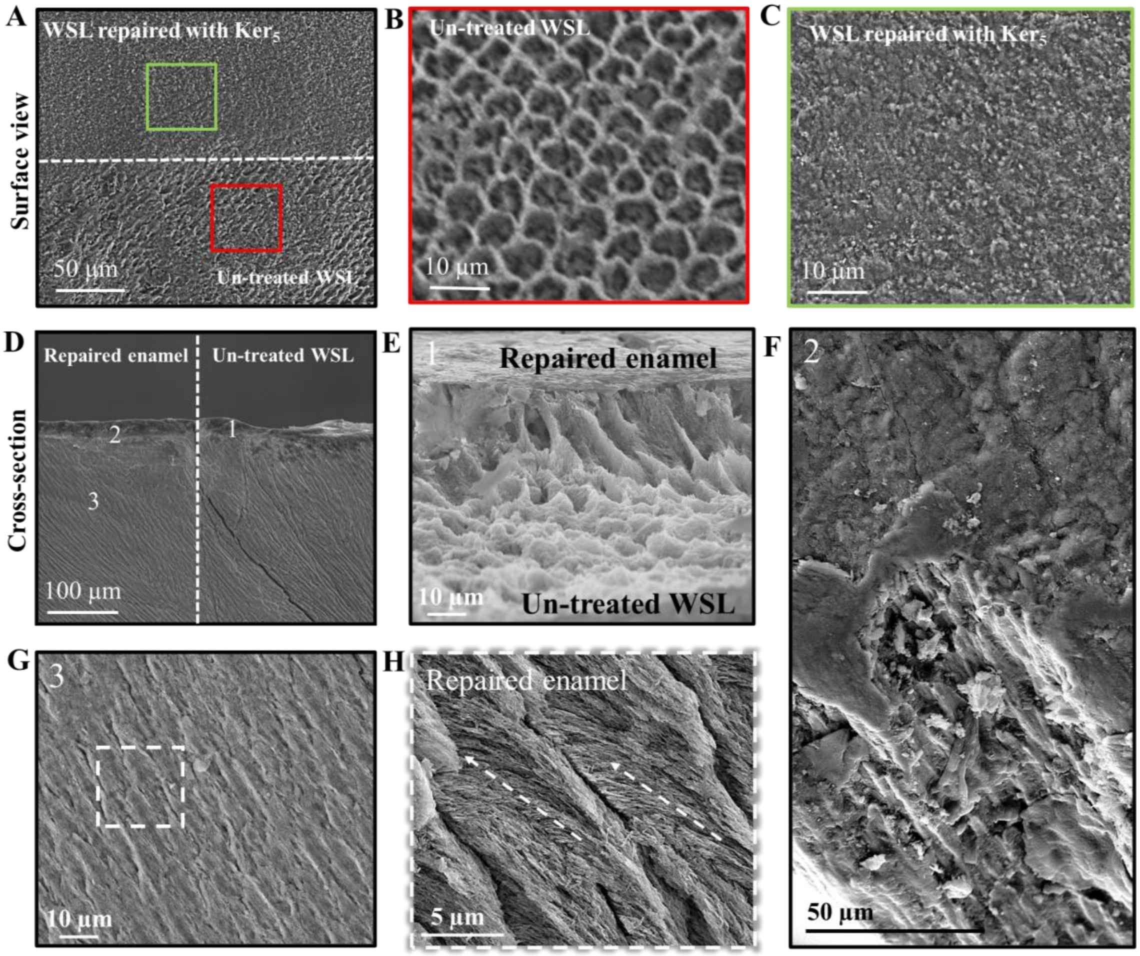

Supplementary Figure 18: Surface characterization of treated enamel lesions incubated in Artificial saliva.

A Interface between WSL (bottom) and lesion treated with keratin (Top). **B** Surface view of WSL before treatment demonstrating the porous enamel due to inducing WSL. **C** Surface view of the WSL treated with keratin showing the repaired enamel**. D** Cross section of a WSL window; demonstrating the interface between the newly formed enamel repaired with keratin of about 30-40μm thickness (left) and the lost prisms in the WSL (right) marking 3 different regions 1, 2, and 3; **E** Shows high magnification of the interface between treated and un treated at the enamel surface, **F** The repaired enamel at the surface, and **G** Cross-sectional view of the repaired enamel prisms and inter-prisms in the WSL side at a depth about 100 μm away from the enamel surface. **H** High magnification of the WSL treated with keratin demonstrating the repaired layer of the newly formed

crystals between the native enamel filling the gaps, dotted arrows show the direction of the repaired enamel crystals.

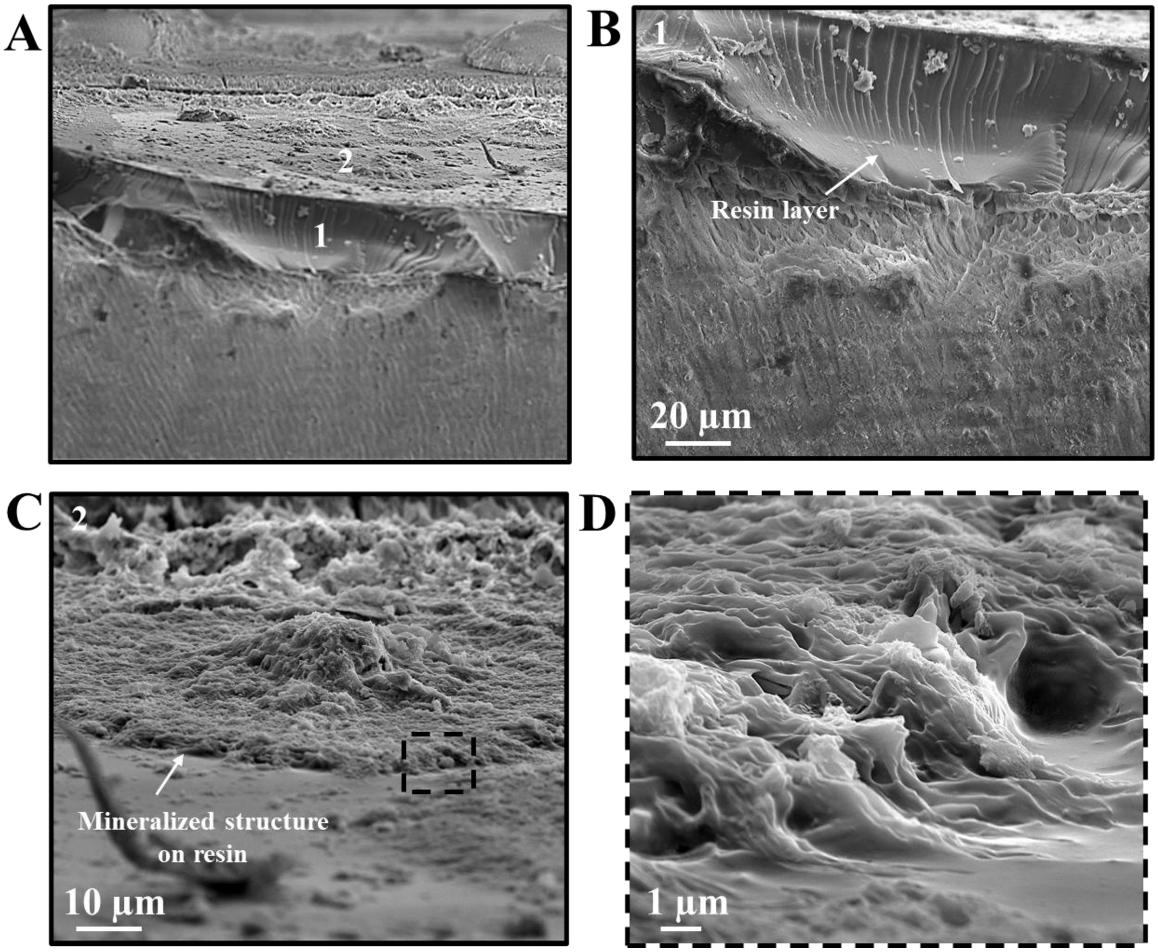

Supplementary Figure 19: Surface characterization of enamel lesions treated with resin infiltrant.

A, B Demineralized resin treated lesions revealing a resin coating on top of native enamel.

C, D Mineralized precipitates of disorganized pattern on top of the resin coating.

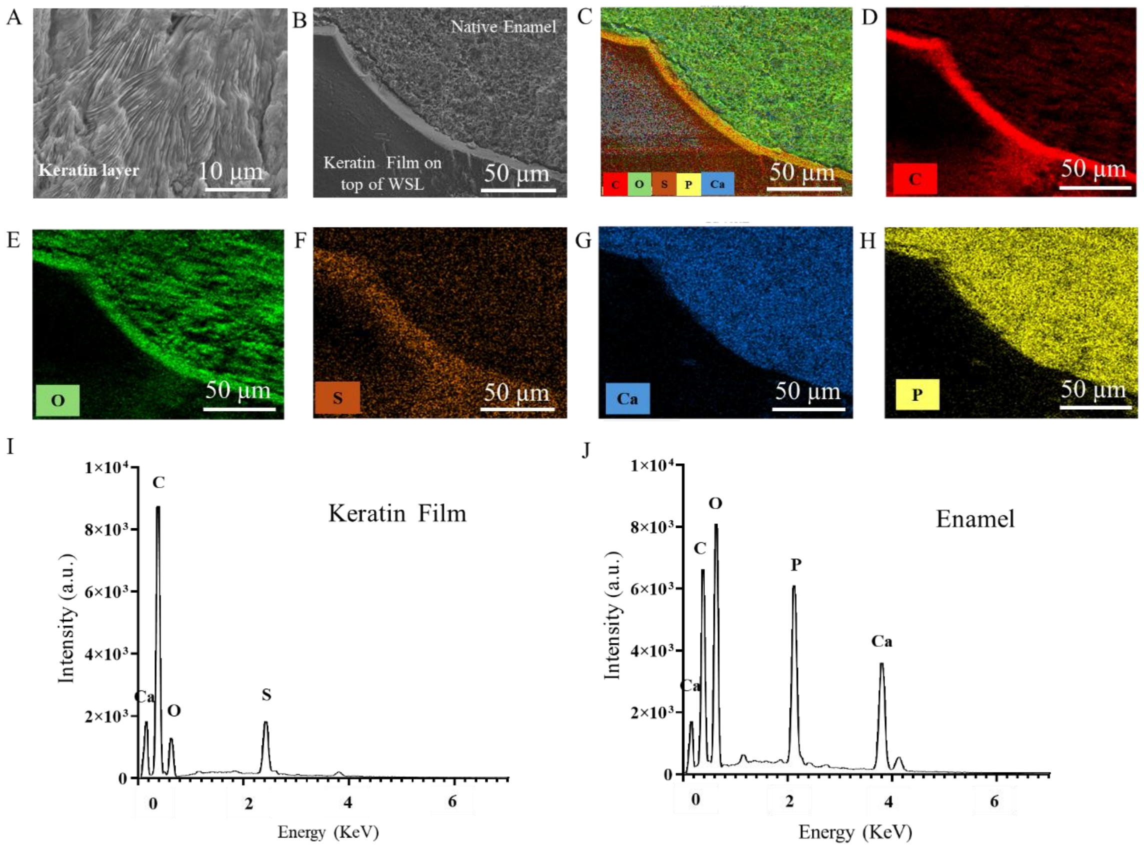

Supplementary Figure 20: Surface characterization of keratin film in UPW.

SEM of the keratin film introduced over WSL without mineralization demonstrating: **A** The keratin layer in cross-section penetrating the WSL to a great depth. *Inset showing low magnification of the keratin layer. **B** The unmineralized keratin film meeting the enamel and both showing different topographies. **EDX mapping image shows elements of each half representing: C** Layered image, **D** Carbon, **E** Oxygen, **F** Sulfur, **G** Calcium, **H** Phosphorous. **Spectra of EDX analysis of the**: **I** keratin film showing high sulfur and carbon. **J** Enamel WSL showing increased calcium and phosphorous.

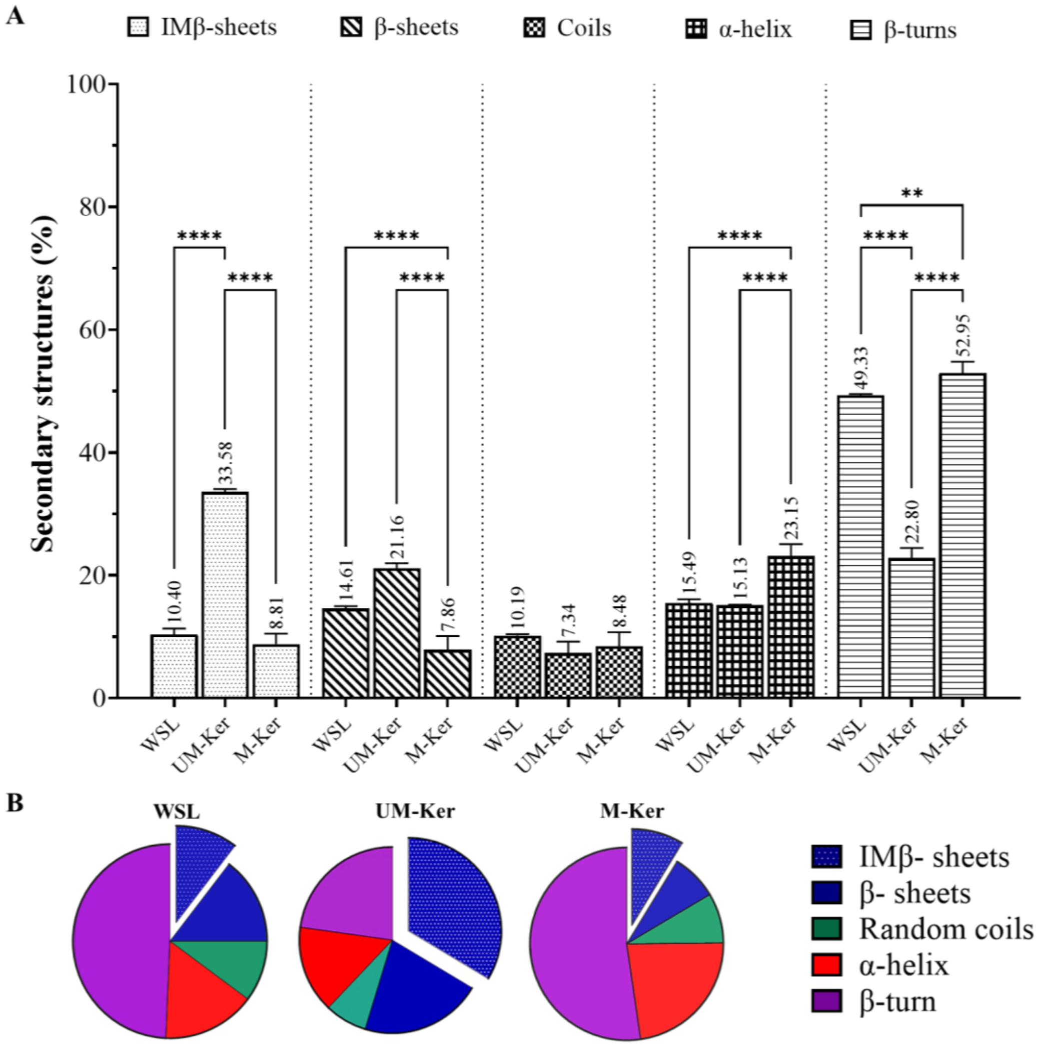

Supplementary Figure 21: FTIR spectroscopy analysis of WSL pre- and post-mineralization.

FTIR Deconvolution analysis demonstrates the secondary structural changes associated with keratin-enamel interactions. **A** The bar charts demonstrate that WSLs exhibited high content of β-turns, while unmineralized keratin exhibited a substantial increase in β-sheet structures. However, upon incubation of keratin-treated lesions in a mineralization solution, notable increase in β-turn structures and α-helix content accompanied by a reduction in β-sheet structures was observed suggesting an interaction between keratin and the minerals provided by the mineralization solution. **B** Pie charts demonstrating the secondary structures percentages of WSLs before remineralization, un-mineralized keratin, and mineralized WSL with keratin, respectively. * Indicates significant difference, where significance at $p < 0.01$, n=3.

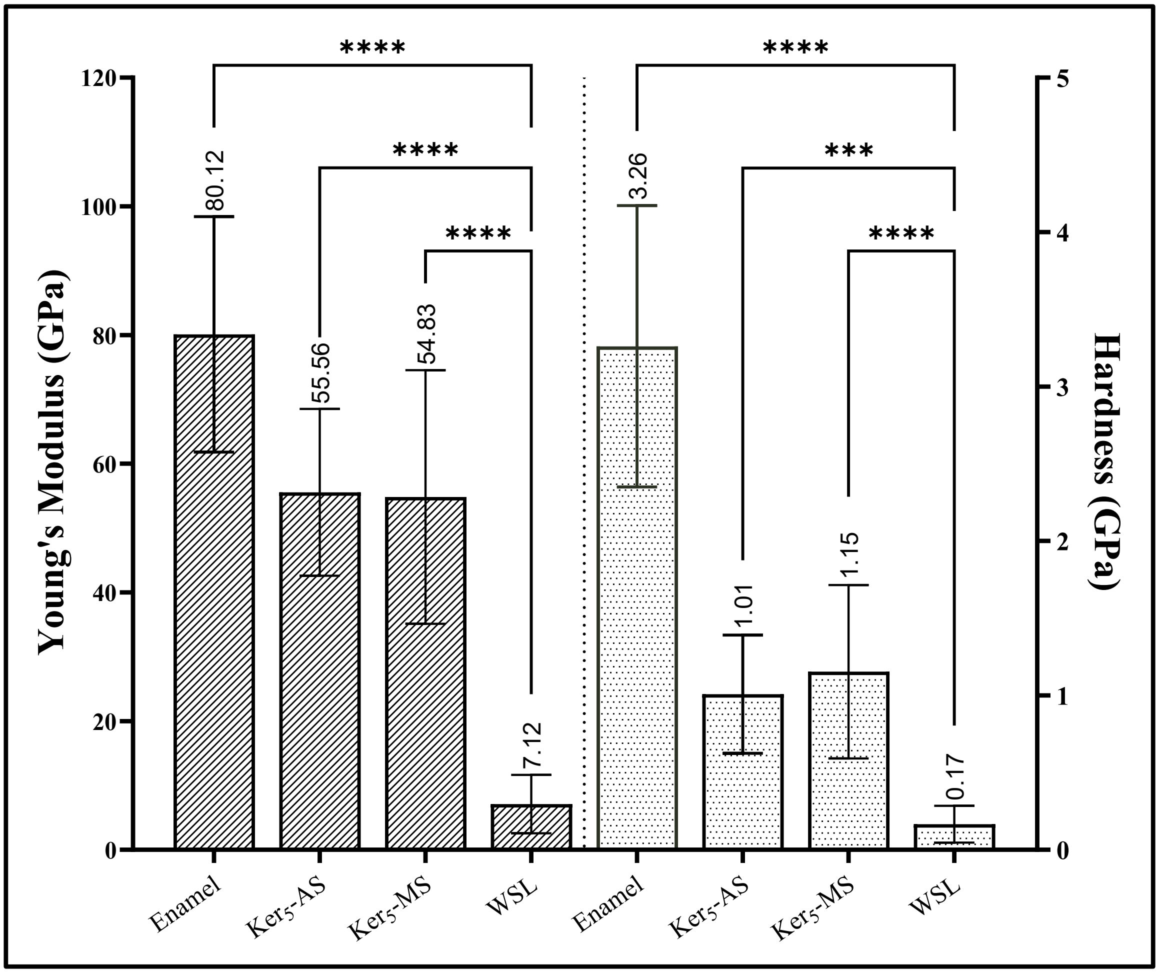

Supplementary Figure 22: Bulk nanoindentation analyses.

Bulk (subsurface) nanoindentation measurements (Mean ± S.D.) of the WSLs subsurface before and after treatments (n=6). The subsurface nanoindentation measurements followed the same trend as the surface ones. The WSLs treated with keratin and incubated in either MS or AS showed significant improvements in the elastic modulus and hardness of the repaired enamel subsurface compared to WSL before treatment. This confirms the infiltration of the keratin within the lesion pores and its ability to remineralize WSL. MS: mineralization solution, AS: artificial saliva. * Indicates significant difference, where significance at p <0.01, n=3.

References

[1] J.K. Eng, A.L. McCormack, J.R. Yates, An approach to correlate tandem mass spectral data of peptides with amino acid sequences in a protein database, J Am Soc Mass Spectrom 5 (1994) 976–989. https://doi.org/10.1016/1044-0305(94)80016-2.

[2] L. Whitmore, B.A. Wallace, DICHROWEB, an online server for protein secondary structure analyses from circular dichroism spectroscopic data, Nucleic Acids Res 32 (2004) W668–W673. https://doi.org/10.1093/nar/gkh371.

[3] G.L. Ellman, Tissue sulfhydryl groups, Arch Biochem Biophys 82 (1959). https://doi.org/10.1016/0003-9861(59)90090-6.

[4] O. Bunk, M. Bech, T.H. Jensen, R. Feidenhans'l, T. Binderup, A. Menzel, F. Pfeiffer, Multimodal x-ray scatter imaging, New J Phys 11 (2009) 123016. https://doi.org/10.1088/1367-2630/11/12/123016.

[5] J. Zhang, R.J.M. Lynch, T.F. Watson, A. Banerjee, Remineralisation of enamel white spot lesions pre-treated with chitosan in the presence of salivary pellicle, J Dent 72 (2018). https://doi.org/10.1016/j.jdent.2018.02.004.

[6] M. Eisenburger, M. Addy, J.A. Hughes, R.P. Shellis, Effect of Time on the Remineralisation of Enamel by Synthetic Saliva after Citric Acid Erosion, Caries Res 35 (2001) 211–215. https://doi.org/10.1159/000047458.

[7] H. Chen, K. Sun, Z. Tang, R. V. Law, J.F. Mansfield, A. Czajka-Jakubowska, B.H. Clarkson, Synthesis of Fluorapatite Nanorods and Nanowires by Direct Precipitation from Solution, Cryst Growth Des 6 (2006) 1504–1508. https://doi.org/10.1021/cg0600086.

[8] S. Habelitz, S.J. Marshall, G.W. Marshall, M. Balooch, Mechanical properties of human dental enamel on the nanometre scale, Arch Oral Biol 46 (2001) 173–183. https://doi.org/10.1016/S0003-9969(00)00089-3.
